# Cotton leaf curl Multan virus differentially regulates innate antiviral immunity of whitefly (*Bemisia tabaci*) vector to promote cryptic species-dependent virus acquisition

**DOI:** 10.3389/fpls.2022.1040547

**Published:** 2022-11-14

**Authors:** Tahir Farooq, Qi Lin, Xiaoman She, Ting Chen, Zhenggang Li, Lin Yu, Guobing Lan, Yafei Tang, Zifu He

**Affiliations:** Guangdong Provincial Key Laboratory of High Technology for Plant Protection, Plant Protection Research Institute, Guangdong Academy of Agricultural Sciences, Guangzhou, China

**Keywords:** begomovirus, innate immunity, *Cotton leaf curl Multan virus*, *Bemisia tabaci*, virus acquisition

## Abstract

Begomoviruses represent the largest group of economically important, highly pathogenic, DNA plant viruses that contribute a substantial amount of global crop disease burden. The exclusive transmission of begomoviruses by whiteflies (*Bemisia tabaci*) requires them to interact and efficiently manipulate host responses at physiological, biological and molecular scales. However, the molecular mechanisms underlying complex begomovirus-whitefly interactions that consequently substantiate efficient virus transmission largely remain unknown. Previously, we found that whitefly Asia II 7 cryptic species can efficiently transmit *cotton leaf curl Multan virus* (CLCuMuV) while MEAM1 cryptic species is a poor carrier and incompetent vector of CLCuMuV. To investigate the potential mechanism/s that facilitate the higher acquisition of CLCuMuV by its whitefly vector (Asia II 7) and to identify novel whitefly proteins that putatively interact with CLCuMuV-AV1 (coat protein), we employed yeast two-hybrid system, bioinformatics, bimolecular fluorescence complementation, RNA interference, RT-qPCR and bioassays. We identified a total of 21 Asia II 7 proteins putatively interacting with CLCuMuV-AV1. Further analyses by molecular docking, Y2H and BiFC experiments validated the interaction between a whitefly innate immunity-related protein (BTB/POZ) and viral AV1 (coat protein). Gene transcription analysis showed that the viral infection significantly suppressed the transcription of *BTB*/*POZ* and enhanced the accumulation of CLCuMuV in Asia II 7, but not in MEAM1 cryptic species. In contrast to MEAM1, the targeted knock-down of *BTB*/*POZ* substantially reduced the ability of Asia II 7 to acquire and accumulate CLCuMuV. Additionally, antiviral immune signaling pathways (*Toll*, *Imd*, *Jnk* and *Jak*/*STAT*) were significantly suppressed following viral infection of Asia II 7 whiteflies. Taken together, the begomovirus CLCuMuV potentiates efficient virus accumulation in its vector *B. tabaci* Asia II 7 by targeting and suppressing the transcription of an innate immunity-related *BTB*/*POZ* gene and other antiviral immune responses in a cryptic species-specific manner.

## Introduction

Inter-host movement of the viruses causing potential damages to humans, animals and plants is predominantly governed by arthropod vectors (Gray and Banerjee, 1999; Eigenbrode et al., 2018). Given that insect-borne transmission of plant pathogens (fungi, bacteria, phytoplasmas and viruses) mainly depends on the behavior and abundance of their vectors; these pathogens, therefore, have been selected to directly and/or indirectly influence their vectors to enhance transmission efficiency. This pathogen-induced vector manipulation might involve combinations of sophisticated effects on the behavior and performance of the vector combined with dynamics of pathogen acquisition that ultimately promote the pathogen spread (Eigenbrode et al., 2018). Of ~ 1,100 known plant-infecting viruses, a majority (over 75%) is exclusively vectored by diverse insect species, mainly by whiteflies (Hogenhout et al., 2008; Ng and Zhou, 2015). As of 2020, begomoviruses (family *Geminiviridae*) constitute the largest group of over 445 single-stranded DNA (ssDNA) viruses that are known to cause potential crop damages worldwide (Walker et al., 2020). These viruses are transmitted by *Bemisia tabaci* species complex in a persistent circulative manner (Harrison and Robinson, 1999; Rosen et al., 2015).

The circulative journey of plant viruses in their vectors occurs in an interactive yet coordinated manner to facilitate successful viral transmission. Concurrently, the viral infection might stimulate the immune responses from its vector (Gray and Banerjee, 1999; Power, 2000). Following the acquisition by insect vectors during feeding, persistent, circulative viruses then move through the alimentary canal of their vector, infect epithelial cells of the gut and are subsequently released into the hemolymph where they can spread to other tissues. Eventually, by infecting the salivary glands, they are released into the salivary duct, carried in the saliva followed by transmission to new/uninfected plants during feeding of the viruliferous vector (Hogenhout et al., 2008). The extent of success and viral transmission efficiency involves specific interactions between known viral factors and unknown vector proteins that ultimately regulate the viral transport in and out of the vector tissue and enable the virus to overcome different types of vector’s immune responses (Gray and Banerjee, 1999; Power, 2000; Schwarz et al., 2014).

To date, AV1 or coat protein (CP) is the only known structural component of begomoviruses that regulates their movement in the insect vector (Harrison et al., 2002). It has been demonstrated that replacement or mutation of the AV1 greatly alters the vector specificity towards geminiviruses transmission (Briddon et al., 1990; Pan et al., 2020). However, until now, only a limited number of whitefly proteins have been reported to interact with AV1. For instance, vesicle-associated membrane protein-associated protein B (VAPB) and heat shock protein-70 (HSP-70) have been reported to exhibit inhibitory functions in viral transmission (Götz et al., 2012; Zhao et al., 2019). On the contrary, GroEL protein of the secondary endosymbionts *Arsenophonus* or *Hamiltonella* prevents the viral degradation in the hemolymph of its vector (Gottlieb et al., 2010; Rana et al., 2012). Furthermore, BtPGRP, a peptidoglycan recognition protein is related to various immune responses of the whitefly and plays role in the begomoviral acquisition (Wang et al., 2016). Recently, a study has elaborated on the inhibitory role of immunity-related tumorous imaginal discs (TIDs) in begomoviral acquisition (Zhao et al., 2020).

The BTB (BR-C, ttk and bab) or POZ (Poxvirus and zinc finger) domain was first identified as a conserved motif in the *Drosophila melanogaster* (Lours et al., 2003). The BTB/POZ is evolutionarily conserved and a protein-protein interaction domain frequently found at the N-terminus of nuclear DNA-binding or actin-binding proteins (Albagli et al., 1995). Proteins containing BTB/POZ domain are known to be associated with versatile cellular functions including protein-protein interactions (PPIs), cytoskeleton dynamics, transcriptional regulation, ubiquitination of proteins and ion channel assembly/gating (Stogios et al., 2005). Nevertheless, the function of BTB/POZ domain-containing proteins in innate immune responses is still less studied. For instance, it has been demonstrated that *ZBTB20* (a member of the BTB/POZ family) enhances toll-like receptor (TLR)-mediated immunity by suppressing the transcription of *IκBα* gene in response to invading pathogens (Liu et al., 2013). Earlier, it was demonstrated that a *HaBBP* gene containing BTB, BTB and carboxyl-terminus kelch repeats (BACK) and PAM, highwire and RPM (PHR) domains is involved in regulating the innate immunity of insects (*Helicoverpa armigera*) *via* 20-hydroxyecdysone (20E) signaling pathway (Wang et al., 2011). Likewise, a recent study has shown that in humans, a nucleus accumbens-associated 1 or NAC1 protein (of BTB/POZ family) is associated with RIG-I-like receptors (RLRs)-mediated innate immunity and stimulates the antiviral signaling in response to multiple RNA viruses (Xia et al., 2019). Additional experimental evidence has reported the discovery of a novel BTB domain-containing protein (SANBR) which acts as a negative regulator of the class switch recombination (CSR) (Zheng et al., 2021); a mechanism that enables B-cells to switch the production of IgD/IgM to generate antibodies of other isotypes (IgA, IgE or IgG) (Chaudhuri and Alt, 2004). The findings of another recent study demonstrate that NbBTB (a BTB/POZ domain-containing protein) affects the accumulation of avirulence effectors and subsequently, negatively modulates basal defense and effector-triggered immunity (ETI) in plants (Zhao et al., 2022).

*Cotton leaf curl Multan virus* (CLCuMuV) is a highly recombinant begomovirus capable of causing potential qualitative and quantitative losses to the cotton industry. Our recent findings demonstrate that the incessant resurgence of CLCuMuV strains in the major cotton-producing countries (China, India and Pakistan) is attributed to their rapid evolvability enabling their quick adaptation to a variety of ecosystems (Farooq et al., 2021). In China, both whitefly cryptic species (Asia II 7 and MEAM1) have been reported to infest the diseased plants exhibiting symptoms of CLCuD (Ting et al., 2016). These biotypes (cryptic species) can easily be distinguished by analyzing their *mtCOI* gene sequences (Chen et al., 2002). Also, we observed that *B. tabaci* cryptic species Asia II 7 is an efficient vector of CLCuMuV infecting malvaceous crops in China (Chen et al., 2019). Thus, we speculate that rapid evolutionary dynamics together with efficient vector acquisition are the leading causes of CLCuMuV resurging outbreaks in the main cotton-producing regions of the world. Recently, researchers have identified several candidate whitefly proteins that interact with CLCuMuV-AV1, including a vacuolar protein sorting-associated protein (Vps) twenty associated 1 (Vta1). Reportedly, *Vta1* of Asia II 1 whiteflies exhibited a strong interaction with CLCuMuV-AV1 as compared to that of MEAM1. Moreover, in contrast to MEAM1, RNAi-mediated knock-down of *Vta1* gene in Asia II 1 whiteflies significantly increased the acquisition and transmission of CLCuMuV (Chi et al., 2021). Although this is an interesting study that highlights the role of *Vta1* in the whitefly-borne transmission of CLCuMuV, the molecular mechanisms governing the efficient transmission of CLCuMuV by Asia II 7 largely remain in the shadow.

Here, we employed a combinatorial approach including yeast two-hybrid assay, bioinformatics, BiFC and RNA interference to elucidate the potential mechanism/s governing efficient transmission of CLCuMuV by whitefly cryptic species, Asia II 7. Numerous whitefly proteins were found to interact with AV1 (CP) of the CLCuMuV. The interactions were further validated followed by the functional analysis of the target whitefly genes. Based on the experimental evidence, we propose that AV1 of the CLCuMuV directly interacts with a whitefly BTB/POZ domain-containing protein, regulates its transcription in a cryptic species-dependent manner and enhances viral acquisition by successful suppression of the immunity in Asia II 7. Of several additional mechanisms involved in the whitefly-mediated begomoviral transmission (Pan et al., 2017; Zhao et al., 2019; Zhao et al., 2020; Wang et al., 2020; Zhao et al., 2020; Ghosh and Ghanim, 2021), our proposed mechanism will facilitate understanding the efficient cryptic species-specific transmission of CLCuMuV.

## Materials and methods

### Insect rearing, plants and virus source maintenance

*B. tabaci* Asia II 7 and MEAM1 populations were maintained on cotton (cv. Zhongmian 43 seedlings) not infected by CLCuMuV inside insect-free cages as this cultivar is a non-host plant of CLCuMuV. The cotton plants were replaced every 30 days by a batch of fresh plants to facilitate whiteflies maintenance. The purity of *B. tabaci* Asia II 7 (biotype CV) and MEAM1 (biotype B) cryptic species cultures was confirmed every 3-5 generations by using mitochondrial cytochrome oxidase I (*mtCOI*) gene-specific primers mtCOI-C1- J- 2195/mtCOI-TL2N- 3014 (**Supplementary Table S1**). For cryptic species purity analysis, 10-15 individual whiteflies were collected, subjected to PCR and subsequently confirmed by sequencing. *Nicotiana benthamiana* wild type (WT) and transgenic (H2B) plants were grown in a substrate mixture containing black soil, artificial soil, perlite and vermiculite (2:1:2:2), in a growth chamber with 14-h light/10-h dark photoperiod at a temperature of 25-27 °C and relative humidity of 60%. The CLCuMuV was maintained by infiltrating the *G. hirsutum* cv. Xinhai 21 at 2-3 true-leaf stage with agrobacterium GV3101 strain cultures harboring CLCuMuV DNA-A (KP762786) and betasatellite (KP762787). The presence of CLCuMuV in plants and insects was confirmed by using specific primers CLCuMuV-CL F/R & CLCuMuB-beta-F/R (**Supplementary Table S1**). For insect transmission assays, *G. hirsutum* cv. Xinhai 21 plants at 3-5 leaf stage were used.

### Cloning, self-activation and functional assay of bait vector

The full-length coding sequence of CLCuMuV-AV1 was PCR-amplified from plasmid harboring CLCuMuV DNA-A (KP762786) and ligated into *SfiI*-digested bait vectors pBT3SUC and pBT3STE (Dualsystems Biotech). The recombinant plasmids were transformed into *Saccharomyces cerevisiae* strain NMY51 and the expression of CLCuMuV-AV1 was subsequently verified by western blot analysis using anti-AV1 antibodies. Verification of the bait-self activation and functional analysis was performed using different combinations of activation/binding (AD/BD) domain-containing plasmids on different nutritionally selective media. The specific details on different combinations of AD/BD plasmids and types of selective media used for this purpose are described in the **Supplementary Table S2**. Primers used for cloning and construction of bait plasmids are listed in the **Supplementary Table S1**.

### Yeast two-hybrid library construction and preliminary screening

The AV1-interacting Asia II 7 proteins were identified by using DUALmembrane Starter Kit (Dualsystems Biotech) following the manufacturer’s protocol. The cDNA of Asia II 7 was constructed into *SfiI*-digested prey vector, pPR_3_N. The cDNA quality was evaluated as per instructions provided in the kit. Subsequently, the cDNA library was transformed into yeast cells containing pBT_3_STE-AV1. Yeast clones were selected on synthetic defined (SD) media lacking *Trp* and *Leu* (SD-TL) and *Trp*, *Leu* and *His* (SD-TLH) containing 5 mM 3-amintotrizole (3-AT). Subsequently, to verify the interactions, yeast cells were resuspended in 1% NaCl (with OD_600_ adjusted to 1) and restreaked on SD media lacking *Trp*, *Leu*, *His* and *Ade* (SD-TLHA) supplemented with 5 mM 3-amintotrizole (3-AT). Additionally, the *β*-galactosidase activity in yeast cells was monitored to evaluate the preliminary interactions. At the final step, plasmids from yeast cells were recovered and transformed into *Escherichia coli* (DH-5α strain) followed by sequencing. To identify the putative AV1-interacting proteins, the obtained sequences were subjected to BLAST analysis (https://blast.ncbi.nlm.nih.gov/Blast.cgi).

### Bioinformatics analysis of AV1-interacting proteins

The AV1-interacting whitefly proteins identified from Y2H screening were subjected to functional annotation using Blast2GO software available online at (https://www.blast2go.com/). Full-length amino acid sequences were used to perform these analyses. For query proteins, analysis of the metabolic pathways was conducted by using the online BlastKOALA tool in the Kyoto Encyclopedia of Genes and Genomes (KEGG) database (https://www.kegg.jp/blastkoala/).

### *In silico* protein-protein interaction and prediction of binding residues

To determine the potential binding sites of the target proteins (BTB/POZ and CLCuMuV-AV1), binding site prediction tools including Non-partner-specific (NSP)-HomPPI (Xue et al., 2011), PredictProtein (Yachdav et al., 2014), and Protein-protein interaction Sites prediction serVER (PSIVER) (Murakami and Mizuguchi, 2010) were employed. Further, a sequence-based method, PPA-Pred was used to identify interactions in terms of ΔΔG/binding affinity (changed in Gibbs free energy) (Yugandhar and Gromiha, 2014). Finally, based on the modeled structures, DoGsiteScorer (Volkamer et al., 2012), was used to find putative binding pockets in the target proteins.

### Validation of individual protein interactions in yeast

Full-length coding sequences of the AV1-interacting genes identified in preliminary Y2H screening were amplified and cloned into bait vector pPR_3_N following the above-mentioned protocol. Validation of interactions between AV1 and whitefly candidate genes was carried out by transforming the yeast cells followed by their growth on nutritionally selective media following procedures mentioned earlier. The primers used for the construction of BTB/POZ prey vector are listed in the **Supplementary Table S1**.

### *Agrobacterium*-mediated transient expression of proteins in *N. benthamiana*

All plasmid constructs containing target ORFs were chemically transformed into *Agrobacterium tumefaciens* GV3101 cells with pSoup vector. To select the positive clones, colony PCR was done by testing agrobacterium colonies growing on LB medium supplemented with kanamycin and rifampicin antibiotics. The positive clones were separately inoculated to LB media with appropriate antibiotics and incubated at 28 °C until achieved optimum OD_600_. The bacteria were pelleted and resuspended in the infiltration buffer (10 mM MES, 10 mM MgCl_2_, 150 µM Acetosyringone) followed by OD_600_ adjustment. As for BiFC, equal volumes of agrobacteria containing pSPYCE, pSPYNE and p19 silencing suppressor were mixed with OD_600_ being 0.5, 0.5 and 0.3, respectively. The lower epidermis of *N. benthamiana* plants was infiltrated using a 2 mL needleless syringe at 5-6 fully-expanded leaf stage.

### Protein structure and phylogenetic analysis

The BTB/POZ amino acid sequence (XP_018901333.1) identified from the Y2H screening assay was aligned with 80 closely related proteins (**Supplementary Table S3**) from different insect species. Multiple sequence alignments (MSA) were performed using ClustalW. The phylogenetic tree was constructed by the maximum likelihood (ML) method using MEGA X program (Kumar et al., 2018). The bootstrapping consisted of 1000 replicates to support the internal nodes of the tree. The grouping of BTB/POZ-related proteins from different insects has been represented by different colors. Prediction of the conserved protein domains was performed using the conserved domain database (CDD) tool available online at NCBI database (https://www.ncbi.nlm.nih.gov/Structure/cdd/wrpsb.cgi). The calculation and prediction of the 3D structure of BTB/POZ protein were performed using the molecular biology tool available at (http://molbiol-tools.ca/Protein_tertiary_structure.htm).

### Bimolecular fluorescence complementation (BiFC) assay

The full-length coding sequences of CLCuMuV-*AV1* and *BTB*/*POZ* (XM_019045788.1) were PCR amplified from a plasmid template containing the target sequence and from *B. tabaci* cDNA, respectively. The PCR products were digested with *Spe*I/*Sal*I and cloned into pSPYCE-35S and pSPYNE-35S expression vectors to generate constructs with YFP at C and N-terminus, respectively. The construction of in-frame expression vectors was further verified by DNA sequencing. Subsequently, these constructs were transformed into agrobacterium and co-inoculated to *N. benthamiana* leaves as described above. After infiltration, plants were maintained at 25 °C ± 2 in darkness and expression of YFP was observed at 72 dpi. To observe YFP complementation, lower epidermal cells were imaged by Zeiss LSM 710 confocal microscope following the manufacturer’s protocol. Primers used for BiFC experiment are listed in **Supplementary Table S1**.

### *In vitro* transcription of dsRNA

The target *BTB*/*POZ* and *GFP* gene sequences were amplified from pPR3N and pGDGm plasmids, respectively. The specific primers used for dsRNA synthesis contained the extended sequence of T7 RNA polymerase promoter (5’-TAATACGACTCACTATAGG-3’) at the 5’ end (**Supplementary Table S1**). The amplified PCR products were used as templates to synthesize dsRNA using T7 RiboMAX™ Express RNAi System (Promega, Madison, WI, USA), following the manufacturer’s protocol. Transcription of the sense and anti-sense strands was carried out in the same reaction. The dsRNA was eluted in nuclease-free water and detected in 1.5% agarose gel electrophoresis followed by qualitative and quantitative analysis of dsRNA using NanoDrop 2000. The dsRNA was either used fresh or preserved at -80 °C for downstream experiments. The ds*GFP* was used as the negative control.

### Silencing of *BTB/POZ* in *B. tabaci* by oral administration of dsRNA

Approximately 150 newly-emerged, virus-free whiteflies (Asia II 7 and MEAM1) were collected from cotton plants using plastic tubes (30 _˟_ 100 _mm_). Tube openings were covered with 0.1% DEPC solution-treated parafilm layers to remove any RNase. A mixture containing ~ 300-400 ng/ul dsRNAs (ds*BTB*/*POZ* or ds*GFP*) solution mixed with 15% sucrose was sandwiched between two layers of parafilm. For each treatment, six tubes were used. The tubes were maintained at ~ 25 °C and 65% relative humidity. The mortality of whiteflies post-dsRNA feeding was observed on daily basis. To analyze the RNAi-mediated gene silencing efficiency, *BTB*/*POZ* transcriptional changes in response to dsRNA treatment were measured at 12, 24, 36 and 48 hours post dsRNA feeding. RNA was extracted from whiteflies in replicate groups consisting of 10-15 individuals followed by RT-qPCR analysis. The primers used for the analysis of *BTB*/*POZ* mRNA levels are listed in the **Supplementary Table S1**.

### Nucleic acid extractions for RT-qPCR and qPCR

Total RNA was extracted from virus-free, virus-infected and dsRNA-fed whiteflies by using TRIzol reagent (Life Technologies, Inc., MD, USA). The RNA quantity was analyzed by NanoDrop 2000 spectrophotometer (Thermo Fisher Scientific, CA, USA) and the integrity was analyzed by 0.8% gel electrophoresis. Then, cDNA synthesis was carried out by using HiFiScript gDNA Removal RT Mastermix, CW2020M (CoWin Biosciences, China) as per provided instructions. The cDNA was stored at -80 °C for downstream applications. RT-qPCR was performed using TB Green^®^ *Premix Ex Taq*™ II (Tli RNaseH Plus) (Takara Bio, Inc. Otsu, Japan) following the manufacturer’s protocol. A total of three biological and nine technical repeats were used in each treatment. DNA extraction from plant samples was done using EasyPure Plant Genomic DNA Kit (Transgene Biotech, Beijing, China) following the manufacturer’s directions. DNA from viruliferous, aviruliferous and dsRNA-fed whiteflies was extracted in lysis buffer according to a procedure described earlier (Pan et al., 2017).

### Analysis of *BTB/POZ* transcription after virus acquisition

Newly-emerged whiteflies (Asia II 7 and MEAM1) were collected from cotton plants and released to feed on the CLCuMuV-infected cotton for virus acquisition. Whiteflies feeding on the virus-free cotton plants were used as control. Approximately 90-120 whiteflies were collected from each treatment consisting of 3 biological and 3 technical repeats (9 replications/treatment). Virus-infected whiteflies were collected at 12, 24, 36 and 48 hours post-feeding on the CLCuMuV source. Total RNA was extracted with TRIzol methods followed by cDNA synthesis as described above. The mRNA levels of *BTB*/*POZ* among whiteflies in response to CLCuMuV infection were analyzed by RT-qPCR using 2^−ΔΔCt^ method (Livak and Schmittgen, 2001). The mRNA expression levels were normalized against *β-actin* and *ribosomal protein L29* (RPL29) genes as the internal controls. Additionally, ~8-10 whitefly adults were randomly selected to confirm the presence of virus using CLCuMuV-specific primers. All primers used in this experiment are listed in the **Supplementary Table S1**.

### Virus acquisition assays

To compare the efficiency of CLCuMuV acquisition after feeding on ds*BTB*/*POZ* or ds *GFP*, whiteflies (Asia II 7 and MEAM1) were subjected to dsRNA feeding for 48 hours and then transferred to CLCuMuV-infected cotton plants to acquire the virus. Whiteflies were collected in groups of 10 at 12, 24, 36 and 48 hours after feeding on the virus source. DNA was extracted by homogenizing whiteflies in 100 µl lysis buffer (10 mM Tris, 0.2% gelatin, 50 mM KCl, 0.45% Tween-20, 60 mg/mL proteinase K and NP-40 with pH adjusted to 8.4). Three biological replicates were included followed by quantitative analysis of viral DNA using qPCR. Primers used in this experiment are listed in the **Supplementary Table S1**.

### Statistical analysis

The relative abundance of CLCuMuV in whiteflies, expression levels of *BTB*/*POZ* gene and survival rates were compared by using independent *t*-test analysis. Significant differences were designated based on *P*-values (*P <0.05, **P <0.01 and *** *P <*0.001). All statistical analyses were performed using IBM SPSS Statistics (SPSS Inc., Chicago, USA).

## Results

### Investigation of interactions between CLCuMuV-AV1 and Asia II 7 whitefly proteins

**Figure 1:**
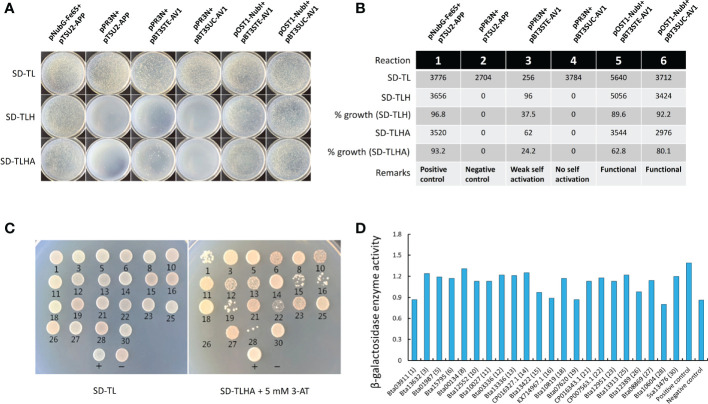
Bait self-activation test and functional analysis and interactions between Asia II 7 proteins and CLCuMuV-AV1. **(A)** Different combinations of plasmids fused with activation or binding domains (AD/BD) were transformed to yeast cells (NMY51) and their growth was monitored on the screening plates containing synthetic defined (SD) media lacking *Trp* and *Leu* (SD-TL) and *Trp*, *Leu* and *His* (SD-TLH) and *Trp*, *Leu*, *His* and *Ade* (SD-TLHA). **(B)** The number of transformants and percentage growth of plasmids on the selective media. Data represents the growth percentage of co-transformed plasmids used as positive control (1), negative control (2), to test self-activation (3-4) and functional activity (5-6). **(C)** Validation of interactions between CLCuMuV-AV1 and 21 Asia II 7 proteins as indicated by the growth of co-transformed yeast cells on synthetic defined media lacking *Trp*, *Leu*, *His* and *Ade* (SD-TLHA) supplemented with 5 mM 3-amintotrizole (3AT) as a result of Ade and *His3* genes activation. Plasmid combination pNubG-Fe65 + pTSU_2_-APP was used as positive control (+) and pPR_3_N + pTSU_2_-APP was used as negative control (-). **(D)** The preliminarily identified interactions were further validated by testing β-galactosidase enzyme as represented by the activation of *LacZ* reporter gene.

Different combinations of plasmids and composition of nutritional selective media used in this experiment are shown in the **Supplementary Table S2**. Results of the bait self-activation and functional analysis test showed that bait (AV1) displayed a strong functional activity in yeast cells with a growth percentage ranging between 62-80 on SD-TLHA media (**Figures 1A, B**). As for the AV1-based screening of Asia II 7 cDNA, a transformation efficiency of 3.91×10^4^/µg was achieved (**Supplementary Figure S1**). Initially, based on the activation of *ADE2* and *HIS3* reporter genes, 30 positive clonal transformants were obtained on SD-TLHA+5mM 3AT media (**Supplementary Figure S2**). After the Y2H assay, a total of 22 clones were obtained on SD-TL media. These clones were further subjected to growth on the SD-TLHA+5 mM 3AT media combined with *β*-galactosidase assay (**Figures 1C, D**). Subsequent analyses based on the activation of *ADE2*, *HIS3* and *LacZ* reporter genes revealed a total of 21 unique AV1-interacting Asia II 7 whitefly proteins (**Table 1**).

**Table 1:** List of putative whitefly proteins interacting with CLCuMuV-*AV1* in Y2H assay.

No.	NCBI accession	Whitefly genome database	Protein functional annotation	Relevant references
1	XM_025948751.1	Bta03911	Chaperone protein dnaj	(Liang et al., 2022)
2	XM_015784844.2	Bta13632	Cytochrome B5	N/A
3	KM821541.1	Bta01987	Cytochrome c oxidase subunit 1	N/A
4	FP100145.1	Bta15795	ATP-dependent RNA helicase	N/A
5	XM_019048796.1	Bta00134	Elongation factor 2	(Li et al., 2009)
6	XM_003557410.4	Bta12552	Thioredoxin	(Saurav et al., 2019)
7	FP099701.1	Bta10027	Unknown protein	N/A
8	XM_015785310.2	Bta03336	Thioredoxin-like protein 1	(Saurav et al., 2019)
9	FP094029.1	Bta13336	Histone H2A	N/A
10	CP016327.1	N/A	Candidatus Portiera aleyrodidarum strain BT-Z1	N/A
11	XM_020323159.1	Bta13422	Tubulin beta-1 chain	(Chee and AbuBakar, 2004)
12	KX714967.1	N/A	*B. emiliae* mitochondrion, partial genome	N/A
13	FP100486.1	Bta10819	Unknown protein	N/A
14	XM_003571129.4	Bta07620	High mobility group protein B2, putative	N/A
15	CP016343.1	N/A	C. Portiera aleyrodidarum strain China 1	N/A
16	CP007563.1	N/A	Candidatus Portiera aleyrodidarum MED (*Bemisia tabaci*) strain BT-Q	N/A
17	XM_020325952.1	Bta12951	Unknown protein	N/A
18	FP100540.1	Bta13313	Eukaryotic translation initiation factor 1A	(Li et al., 2009; Yue et al., 2011; Lin et al., 2012)
19	XM_019045788.1	Bta12389	BTB/POZ domain-containing protein 10	N/A
20	XM_015791561.2	Bta08869	Oligosaccaryltransferase	(Lin et al., 2017)
21	FP096515.1	Ssa13476	CG13675, isoform D	(Mugerwa et al., 2022)

### Functional annotation and metabolic pathways analysis of AV1-interacting proteins

**Figure 2:**
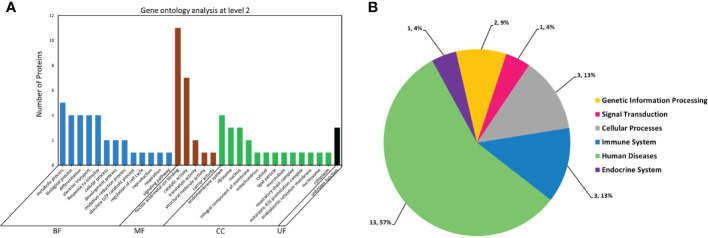
Functional annotation and pathway analysis of 21 whitefly proteins putatively interacting with CLCuMuV-AV1. **(A)** Gene ontology (GO) analysis of 21 whitefly proteins. The protein functional categories associated with the biological function (BF), molecular function (MF), cellular component (CC) and unknown function (UF) were defined using Blast2GO software available online at (https://www.blast2go.com/). **(B)** Metabolic pathway distribution of these proteins was analyzed by using online BlastKOALA tool in Kyoto Encyclopedia of Genes and Genomes (KEGG) database available online at (https://www.kegg.jp/blastkoala/).

GO and KEGG-mediated *in silico* analysis of 21 AV1-interacting proteins classified them into different groups based on biological, molecular or cellular functions. GO analysis identified 13 unique biological processes, 5 molecular functions and 13 categories related to the cell structure with 3 proteins having unknown functions. The highest number (~11) of these proteins was associated with nucleic acid/metal-ion binding molecular function. Whereas, a majority was related to metabolic and biological processes, differentiation, electron transport response to stimuli and catalytic activities. Additionally, a few of them were identified as cellular components associated with the nucleus, ribosome and endomembrane system (**Figure 2A**). Furthermore, KEGG-based pathway analysis divided these proteins into 6 groups associated with genetic information processing, signal transduction, cellular processes, immune system, human diseases and endocrine system (**Figure 2B**). For instance, Bta12389 is a BTB/POZ domain-containing protein-10 which is associated with the molecular function of protein binding (GO:0005515). As mentioned in the introduction, this protein is a protein-binding transcriptional regulator and as a part of the innate immunity system, it possesses antiviral activity. The detailed attributes related to the functional annotation and pathways analysis of 21 AV1-interacting proteins are listed in the **Supplementary Table S4**.

### Protein structure and phylogenetic analysis

**Figure 3:**
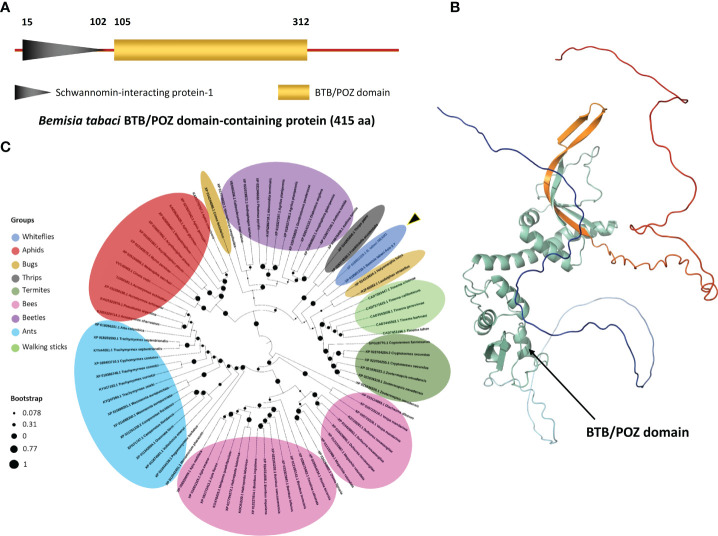
Protein (BTB/POZ) structure and phylogenetic analysis. **(A)** A graphical presentation depicting the type, length and position of the conserved domains as predicted by domain database (CDD) tool available online at NCBI database (https://www.ncbi.nlm.nih.gov/Structure/cdd/wrpsb.cgi). **(B)** Protein 3D structure was calculated and predicted in ChimeraX software (Pettersen et al., 2021). The BTB/POZ domain is highlighted in cyan-green color. **(C)** A phylogenetic tree of BTB/POZ and 80 other closely-related proteins was constructed using MEGA X program. Circular black dots represent the bootstrap values deduced from 1000 replicates. BTB/POZ domain-containing proteins belonging to different insect groups are represented by different colors.

The BTB/POZ prey plasmid sequence obtained from Y2H screen was subjected to BLAST analysis which showed 100% sequence similarity with a 1245 bp long gene sequence (GenBank accession: XM_0109045788.1) coding the BTB/POZ protein (GenBank accession: XP_018901333.1). When BLAST searched on the whitefly genome database (http://www.whiteflygenomics.org/cgi-bin/bta/index.cgi), this protein showed 100% similarity with BTB/POZ domain-containing protein 10 of *B*. *tabaci* MEAM1 (Accession: Bta12389). The BTB/POZ protein is 415 aa long of which, N-terminal contains a Schwannomin-interacting protein-1 domain (15-102 aa) and a conserved BTB/POZ domain (105-312 aa) (**Figure 3A**). The predicted 3D structure of BTB/POZ is shown in the **Figure 3B**, whereas no transmembrane domain was detected in the protein sequence. Phylogenetic analysis of the *B*. *tabaci* BTB/POZ protein and 80 other BTB/POZ domain-containing proteins from different insects showed that it shared a clade with another *B*. *tabaci* BTB/POZ homologous protein (GenBank accession: XP_018901329.1) and displayed 96.51% sequence homology. In the monophyletic group, it appeared to be closely related to BTB/POZ domain-containing protein 10 of *Halyomorpha halys* (GenBank accession: XP_024219824.1) sharing a 75.87% sequence homology (**Figure 3C** and **Supplementary Table S3**).

### *In silico* analysis of protein interactions and prediction of putative binding sites

**Figure 4:**
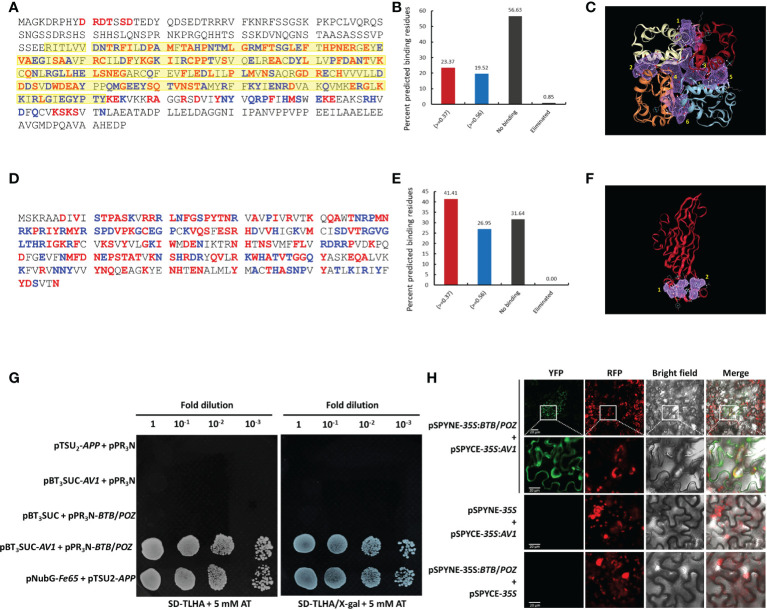
*In silico* prediction and validation of interaction between CLCuMuV-AV1 and whitefly BTB/POZ proteins. **(A, B)** Prediction of putative binding residues of BTB/POZ protein. Yellow highlighted residues denote area covered by BTB/POZ domain **(C)** Six putative binding pockets of the BTB/POZ protein (highlighted in purple-colored grids. **(D, E)** Prediction of putative binding residues of AV1 protein. **(F)** Two putative binding pockets of the AV1 protein (highlighted in purple-colored grids. Red color in A, B, D and E panels indicates the predicted binding residues with optimal threshold (>=0.37) while blue color indicates binding residues with more than 90% specificity (>=0.56). **(G)** Yeast two-hybrid (Y2H) assay was performed using bait (pBT_3_SUC-*AV1*) and prey (pPR_3_N-*BTB*/*POZ*) genes. Yeast cells (NMY51) were co-transformed with bait and prey plasmids and grown on synthetic defined media lacking *Trp*, *Leu*, *His* and *Ade* (SD-TLHA) supplemented with 5 mM 3-amintotrizole with and without X-gal. Serial 10-fold dilutions of yeast cells were made as indicated. Cells co-transformed with pNubG-Fe65 + pTSU_2_-APP served as a positive control, and cells co-transformed with pPR_3_N + pTSU_2_-APP, or with empty vectors (pPR_3_N and pBT_3_SUC) served as negative controls. **(H)** The interaction between CLCuMuV-AV1 and whitefly BTB/POZ protein was confirmed by bimolecular fluorescence complementation (BiFC) assay. Target proteins were cloned into pSPYCE-35S and pSPYNE-35S vectors to generate C and N-terminus yellow fluorescence protein (YFP)-tagged expression cassettes. The YFP complementation in the lower epidermal cells of *N. benthamiana* was observed at 3 dpi using a confocal laser microscope (LSM-710) at 514 nm wavelength. Yellow and red fluorescence protein signals are represented by YFP and RFP, respectively. Scale bars represent 20 µm.

Analysis of the BTB/POZ protein revealed that 97/415 binding residues (23.37%) met the criteria of optimum threshold (>=0.37) while 81/415 (20%) putative binding residues were detected with over 90% specificity (>=0.56). Furthermore, no binding prediction was made for 235/415 (57%) residues (**Figures 4A, B**). Notably, of all positively binding residues, ~80% were detected in the BTB/POZ domain which is well-known for protein interactions. The structure-based computational analysis showed six putative binding pockets (**Figure 4C**) in the BTB/POZ protein. Likewise, analyses were employed to identify putative binding residues and binding pockets in the AV1 protein. We identified 69/256 (27%) residues displaying optimum threshold (>=0.37) while 106/256 (41.4%) binding residues were detected with >90% specificity (>=0.56). Additionally, two putative binding pockets were identified in the CLCuMuV-AV1 protein (**Figures 4D–F**). Further, we employed a sequence-based binding affinity prediction method to determine the possible interaction between BTB/POZ and CLCuMuV-AV1. A highly negative value (ΔΔG = -15.20 kcal/mol) was observed which indicated a strong interaction between two full-length proteins (**Supplementary Figure S3**). The higher negative value of ΔΔG also indicated that the complex between both proteins is more stable.

### Validation of interaction between CLCuMuV-AV1 and BTB/POZ

The candidate whitefly proteins were subsequently analyzed by one-to-one interactions using Y2H system as described in the methods section. Repeated Y2H assays revealed a robust interaction between AV1 (bait) and BTB/POZ (prey) proteins in yeast cells (**Figure 4G**). We took advantage of BiFC to validate this interaction in living cells of *N*. *benthamiana*. For this purpose, lower epidermal cells of *N*. *benthamiana* leaves were observed using a fluorescence microscope at 48-72 hours post-infiltration. The YFP complementation was observed in the cells co-transformed with pSPYNE-BTB/POZ and pSPYCE-AV1 indicating a direct interaction between the two proteins. The YFP signals were mainly distributed in the cell membrane and cell periphery. However, no YFP signals were observed in the control leaves infiltrated with AV1 or BTB/POZ alone, signifying that the interaction between these two proteins is true (**Figure 4H**). Similar results were obtained in the repeated BiFC experiments which further confirmed the specific interaction between CLCuMuV-AV1 and BTB/POZ.

### Transcriptional response of whitefly *BTB/POZ* gene after CLCuMuV acquisition and virus accumulation

**Figure 5:**
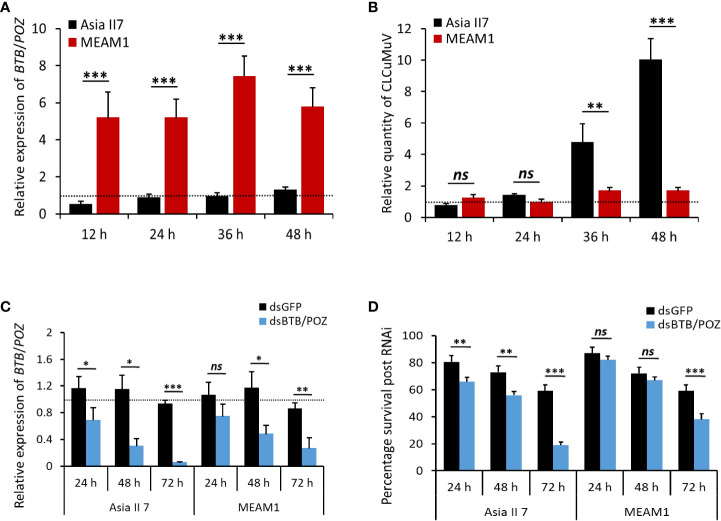
Transcriptional response of *BTB*/*POZ* following CLCuMuV acquisition, expression levels of *BTB*/*POZ* and survival of whiteflies after dsRNA treatment. **(A)** Whiteflies (Asia II 7 & MEAM1 cryptic species) were allowed to feed on the CLCuMuV-infected plants followed by RNA extraction and the mRNA expression levels of *BTB*/*POZ* were monitored at 12 h (*P* = 0.0001), 24 h (*P* = 0.00025), 36 h (*P* = 0.00012) and 48 h (*P* = 0.0001). **(B)** DNA was extracted from the viruliferous whiteflies and the accumulation of viral DNA was assessed at 12 h (*ns* =non-significant), 24 h (*ns*), 36 h (*P* = 0.003748) and 48 h (*P* = 0.000504). Whiteflies were collected in replicate groups (~10 whiteflies/group) and subjected to nucleic acid extraction. Horizontal dashed lines (black) represent the level of control. **(C)** Whiteflies (Asia II 7 & MEAM1 cryptic species) were fed on ds*BTB*/*POZ* and the effect of the interference experiment was analyzed by RT-qPCR assay. Whiteflies fed on ds*GFP* served as control. The relative expression of *BTB*/*POZ* between two cryptic species was compared at different time intervals (24, 48 and 72 h) post-dsRNA feeding. **(D)** The survival rate of the whiteflies in response to interference treatment was also analyzed. Differences between treatments were analyzed by independent *t*-test and the statistical significance was defined as: *P < 0.05, **P < 0.01 and ***P < 0.001. ns, non-significant.

To investigate the patterns of BTB/POZ expression after virus acquisition, *B*. *tabaci* (cryptic species, Asia II 7 and MEAM1) were allowed to feed on the CLCuMuV-infected cotton plants for four different acquisition access periods (12, 24, 36 and 48 hours). Subsequently, the transcription of *BTB/POZ* and virus accumulation in the whole body of whiteflies were analyzed. The RT-qPCR results showed that the expression of *BTB/POZ* immediately increased in MEAM1 at 12 h AAP and followed an increasing trend at the subsequent time points (**Figure 5A**). On the contrary, the expression of *BTB/POZ* was surprisingly lower in Asia II 7 and it appeared similar to that of aviruliferous whiteflies (**Figure 5A**). We further performed qPCR to estimate the levels of virus accumulation and the results revealed that the virus accumulation had no significant difference among both cryptic species (Asia II 7 and MEAM1) at 12 h of AAP (**Figure 5B**). Though the viral DNA was observed to slightly increase in Asia II 7 at 24 h AAP, the difference remained non-significant as compared to MEAM1 (**Figure 5B**). Notably, at 36 h AAP, the CLCuMuV levels in Asia II 7 were significantly elevated and a dramatic increase in the virus accumulation was observed at 48 h AAP. Though CLCuMuV accumulation in MEAM1 slightly increased over time, it remained much lower than that of Asia II 7 (**Figure 5B**).

### RNAi-mediated knock-down of *BTB/POZ* and survival of whiteflies

To optimize the dsRNA-mediated silencing of *BTB/POZ* with maximum efficiency, whiteflies (Asia II 7 and MEAM1) were fed on ds*BTB*/*POZ* (400 ng/ul) for 24, 48 and 72 hours. Whiteflies feeding on ds*GFP* were included as control. RT-qPCR-based analysis of *BTB/POZ* transcription showed that 48 h feeding on ds*BTB*/*POZ* was sufficient to down-regulate the gene expression both in Asia II 7 and MEAM1 (**Figure 5C**). Although the silencing efficiency was much higher in the whiteflies fed on ds*BTB*/*POZ* for 72 h, the percentage of surviving whiteflies was very low (~20%). In general, the overall survival rate post dsRNA feeding remained higher in the MEAM1 whiteflies (**Figure 5D**). These results suggested that the effect and efficiency of *BTB/POZ* silencing were obvious in the Asia II 7 and 48 h feeding on ds*BTB*/*POZ* was optimum to obtain efficient gene silencing and to retain the minimum number of whiteflies required for downstream experiments.

### Effects of *BTB/POZ* silencing on virus acquisition

**Figure 6:**
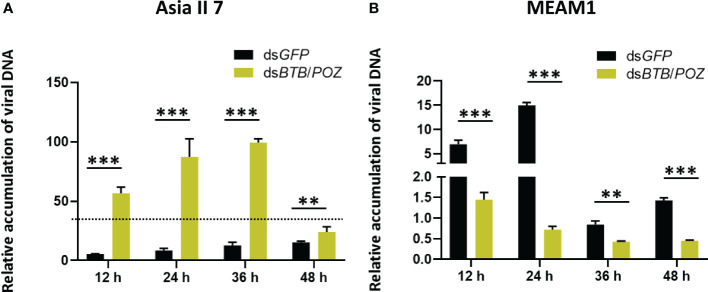
*BTB/POZ* silencing differentially affects virus acquisition/accumulation rates by whiteflies. Post-dsRNA treatment **(A)** Asia II 7 whiteflies were allowed to acquire virus and qPCR-based quantification of the viral DNA was performed at 12 h (*P* =0.00001), 24 h (*P* = 0.00001), 36 h (*P* = 0.00834) and 48 h (*P* = 0.000269). **(B)** Quantification of the viral DNA was also compared between ds*GFP* and ds*BTB*/*POZ*-fed MEAM1 whiteflies at 12 h (*P* =0.00001), 24 h (*P* = 0.00175), 36 h (*P* = 0.00175) and 48 h (*P* = 0.00001). Differences between treatments were analyzed by independent *t*-test and the statistical significance was defined as: **P < 0.01 and ***P < 0.001.

To investigate the role of BTB/POZ in virus acquisition, whiteflies (Asia II 7 and MEAM1) were fed on ds*BTB*/*POZ* and ds*GFP* (400 ng/ul), and followed by 48 h feeding on the CLCuMuV source with AAPs of 12, 24, 36 and 48 h. Whiteflies at the mentioned time intervals were collected in replicate groups and subjected to qPCR analysis. Results showed that silencing of *BTB/POZ* substantially increased the virus accumulation over time in Asia II 7 whiteflies. Although, at 48 hours the CLCuMuV DNA levels were lowered, they remained higher than those of ds*GFP*-fed whiteflies (**Figure 6A**). On the contrary, in MEAM1 whiteflies, the virus accumulation followed a decreasing trend over time as compared to those fed on ds*GFP* (**Figure 6B**). It indicates that *BTB/POZ* silencing in Asia II 7 significantly enhances the efficiency of virus acquisition. Overall, the effects of *BTB/POZ* silencing were more deleterious in Asia II 7 as compared to MEAM1.

### Innate immunity-related pathways in whiteflies are differentially regulated post-CLCuMuV infection

**Figure 7:**
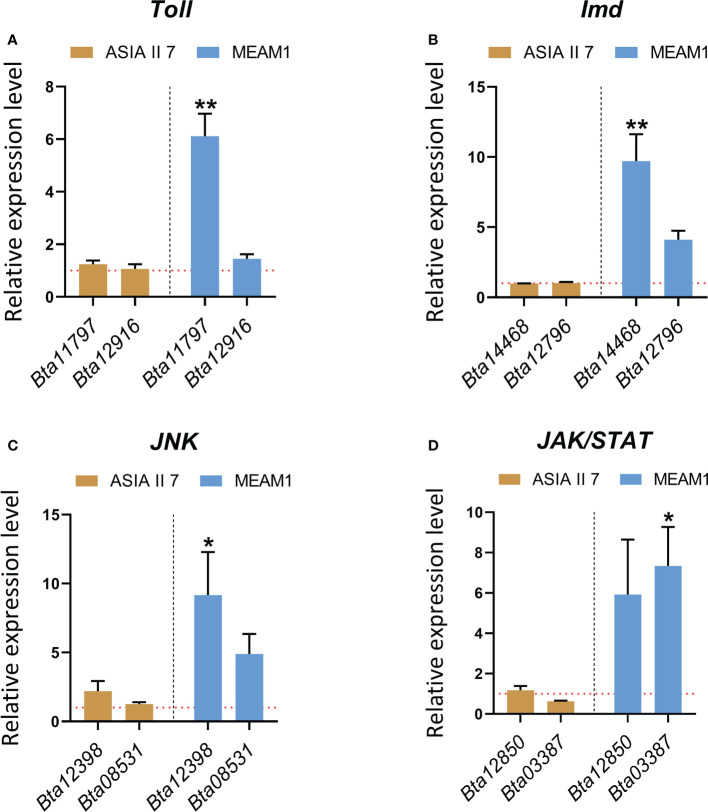
Comparative transcriptional response of whitefly genes involved in the antiviral immune signaling pathways. Whiteflies (Asia II 7 & MEAM1 cryptic species) were allowed to feed on the virus source for 48 hours followed by RNA extraction and RT-qPCR based quantification of the genes involved in **(A)** *Toll* **(B)** *Imd* **(C)** *Jnk* and **(D)** *Jak*/*STAT* antiviral immunity-related pathways. Differences between treatments were analyzed by independent *t*-test and the statistical significance was defined as: *P < 0.05 and **P < 0.01. Horizontal dotted lines (red) denote the relative mRNA expression levels of the target genes in healthy (aviruliferous) whiteflies.

Our finding that selective regulation of immunity-related *BTB*/*POZ* differentially affects the subsequent virus acquisition by whiteflies further led us to hypothesize whether CLCuMuV infection of whiteflies induces/suppresses certain immunity-related pathways in a cryptic species-specific manner. To answer this question, we opted to test the transcriptional responses of eight genes (**Supplementary Table S5**) that are well-known to be associated with major immune signaling pathways (*Toll*, *Imd*, *Jnk* and *Jak*/*STAT*) in the insects (Hillyer, 2016). The results revealed that following a period of 48 h feeding on CLCuMuV-infected plants, the *Toll*, *Imd*, *Jnk* and *Jak*/*STAT* pathways were substantially induced in the MEAM1 whiteflies suggesting an early-stage activation of immune responses to the viral infection (**Figures 7A–D**). Remarkably, the transcriptional levels of immunity-related genes remained significantly lower in the Asia II 7 whiteflies. Although, the immunity pathways appeared to be activated as the mRNA levels of the immunity-related genes remained slightly higher than those in aviruliferous whiteflies; overall, the immune responses remained significantly lower as compared to MEAM1 whiteflies (**Figures 7A–D**).

### Comparative expression analysis of BTB/POZ domain-containing genes among CLCuMuV-infected MEAM1 and Asia II 7 whitefly cryptic species

**Figure 8:**
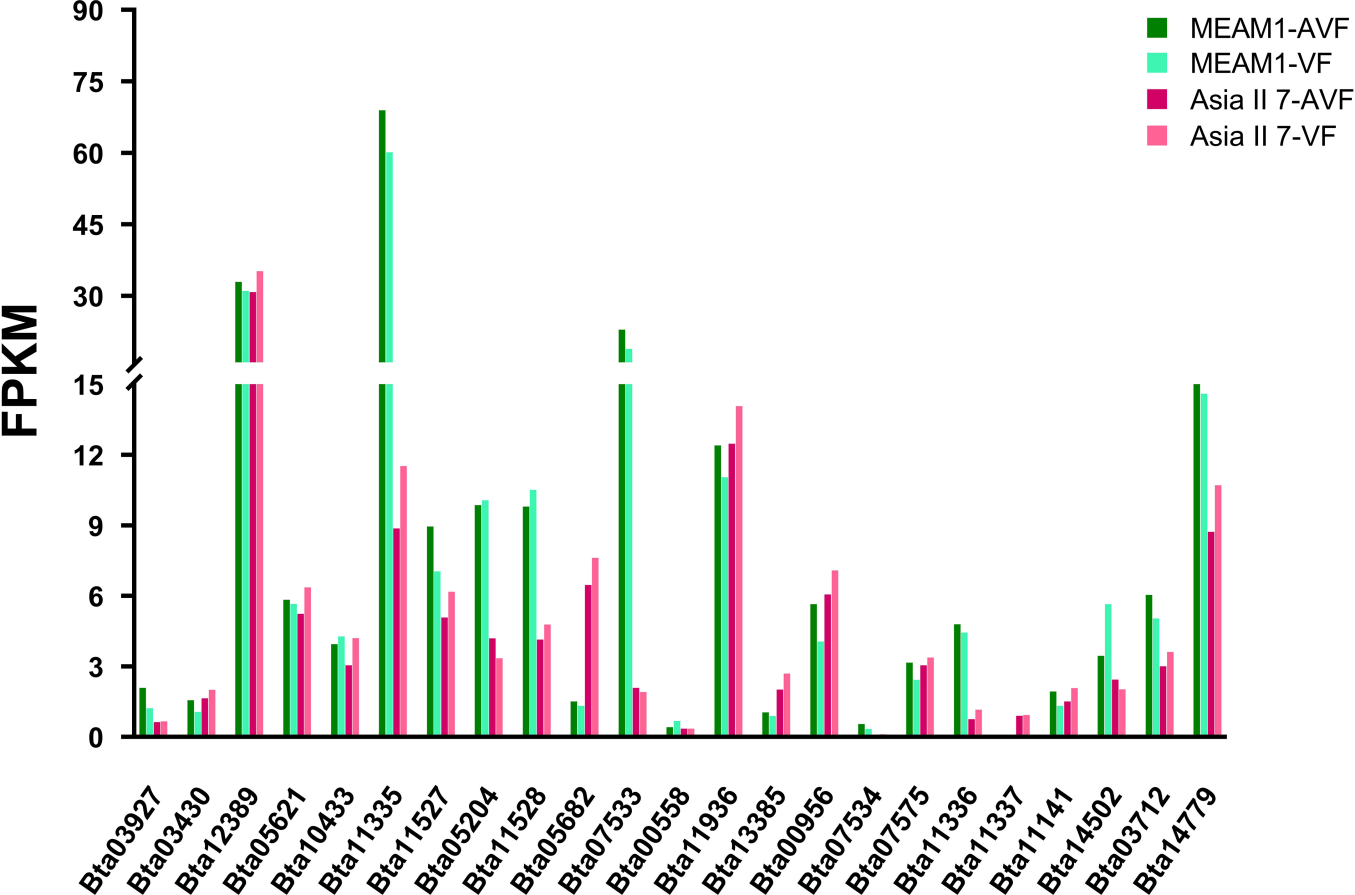
Comparative expression analysis of 23 BTB/POZ domain-containing genes among CLCuMuV-infected Asia II 7 and MEAM1 whiteflies at 12 h AAP.

To further expand our understanding of how other BTB/POZ domain-containing genes are transcriptionally regulated in response to CLCuMuV infection, we performed a transcriptomic analysis of the virus-infected whiteflies (Asia II 7 and MEAM1) at 12 h AAP. Captivatingly, the results of fragments per kilobase of transcripts per million mapped reads (FPKM) analysis demonstrated that of 23 total BTB/POZ domain-containing transcripts, the expression of 13 transcripts was higher among MEAM1 whiteflies as compared with that of Asia II 7 (**Figure 8** and **Supplementary Table S6**). On the contrary, the relative expression of 10 BTB/POZ domain-containing genes was higher in CLCuMuV-infected Asia II 7 whiteflies (**Figure 8** and **Supplementary Table S6**). These results support our aforementioned observation that the transcriptional response of BTB/POZ domain-containing genes is cryptic-species dependent and generally higher among MEAM1 whiteflies post-CLCuMuV infection.

## Discussion

The majority of economically important plant viruses relies on vector-mediated transmission (Whitfield et al., 2015) for dispersal and survival. Plant viruses regulate efficient acquisition and transmission by their insect vectors to establish a successful infection cycle (Dietzgen et al., 2016). Although, the vector-mediated transmission of viruses might appear a simple process of contamination, it is rather a complicated phenomenon involving dynamic interactions between vector and viral proteins. Plant viruses induce several direct and/or indirect effects on their insect vectors that modify their behavior, fitness and life cycle which consequently leads to efficient virus transmission and dissemination (Moreno-Delafuente et al., 2013). In the course of long-term virus-vector co-evolution, the vectors have evolved and continuously impose several constraints on plant viruses to limit their persistent transmission by new vectors (Lefeuvre et al., 2019). To tackle this, on the contrary, viruses have been able to select sophisticated adaptations that improve their efficient transmission by vectors at different scales (Gallet et al., 2018). Thus, the journey of viruses through their insect vectors is mainly driven by specific interactions between viral determinants and vector proteins (Dietzgen et al., 2016). While most of the viral factors have been well-characterized with respect to their vector counterparts (Whitfield et al., 2015), novel receptors of the vectors involved in virus transmission largely remain unknown and specifically, elucidation of the molecular factors regulating the whitefly-mediated transmission of begomoviruses has emerged as a continuously developing research domain (Zhao et al., 2020; Wang et al., 2020; Zhao et al., 2020).

In this study, we took advantage of the split ubiquitin yeast two-hybrid system and identified 21 candidate Asia II 7 whitefly proteins that ostensibly interacted with CLCuMuV-AV1 (**Figure 1** and **Table 1**). GO and KEGG-based analyses further categorized these proteins according to their possible roles in various biological processes and metabolic pathways (**Figure 2**). Next, we constructed the full-length coding sequences of these proteins to verify the preliminarily identified interactions. The results of molecular docking, Y2H and BiFC experiments demonstrated the direct interaction between a zinc finger (BTB/POZ domain-containing) protein (Bta12389) and CLCuMuV-AV1 (**Figure 4**). Often regarded as “born-to-bind” proteins, the BTB/POZ domain-containing proteins constitute a class of transcriptional regulators that are well-known for protein-protein interactions (Perez-Torrado et al., 2006). Additionally, BTB/POZ proteins are associated with diverse biological functions including developmental processes, differentiation, chromatin remodeling and tumorigenesis (Kelly and Daniel, 2006; Lee and Maeda, 2012). Earlier, the BTB/POZ domain has been documented to be associated with biological functions including homeostasis, development, transcriptional repression and neoplasia. Studies on the BTB domain-containing Bcl-6, PLZF-RARα and PLZF have reported that the BTB domain is crucial for dimerization, nuclear localization and transcriptional repression (Dhordain et al., 1995; Dong et al., 1996). Interestingly, few studies suggest the unique roles of BTB/POZ proteins in antiviral (Liu et al., 2013; Xia et al., 2019), proviral (Wang and Zheng, 2021), and innate immune responses against invading pathogens (Wang et al., 2011; Sadler et al., 2015; Loimaranta et al., 2018; Li et al., 2020; Zhang et al., 2021). However, the details regarding their biological functions in virus-vector interactions largely remain understudied.

Given that plant viruses as obligate intracellular parasites, modulate the immune response of their vectors for successful colonization (Wang et al., 2020), we sought to investigate the transcriptional response of *BTB*/*POZ* in response to viral acquisition. Our results demonstrated that the transcription of *BTB*/*POZ* was significantly suppressed in Asia II 7 as compared to the MEAM1 cryptic species in which *BTB*/*POZ* transcription was significantly upregulated post-CLCuMuV acquisition (**Figure 5A**). Based on this observation we hypothesized that the differential expression of *BTB*/*POZ* in whiteflies is directly correlated with their efficiency to acquire CLCuMuV. The results of viral DNA accumulation further corroborated this hypothesis suggesting that CLCuMuV targets the whitefly immunity to enhance CLCuMuV acquisition in a cryptic species-specific manner (**Figure 5B**). Subsequent knock-down of BTB/POZ substantially enhanced the CLCuMuV acquisition and transmission by Asia II 7, however, on the contrary, *BTB*/*POZ* silencing in MEAM1 did not change the CLCuMuV levels (**Figure 6**). While we observed that compared to Asia II 7, MEAM1 exhibits poor efficiency to acquire CLCuMuV, this finding is supported by a recent study that describes the likewise conclusions (Chi et al., 2021).

The viral manipulation of insect immune responses, especially in the context of begomovirus-whitely interactions has been discussed by few studies. In response to viral targeting of the vector immunity at physical, cellular and humoral levels, the insects advantageously regulate innate immune responses to combat these invading pathogens (Lavine and Strand, 2002). For instance, *Bt*PGRP, a whitefly protein with antibacterial properties, has been shown to possess multiple immunity-related functions that affect begomoviral acquisition (Wang et al., 2016). Similarly, a recent finding describes the antiviral role of an immunity-related whitefly protein Tid, in begomovirus acquisition (Zhao et al., 2020). Interestingly, several pieces of evidence suggest that insect-borne viruses can induce the c-Jun N-terminal kinase/JNK signaling pathways of their vectors, which substantially enhances viral replication (Wang et al., 2017; Chowdhury et al., 2020; Ma et al., 2020). Additional data demonstrate that autophagy and apoptosis not only trigger complicated virus-vector interactions but also, govern the whitefly regulation of begomoviral transmission (Wang et al., 2016; Wang et al., 2020; He et al., 2020; Wang et al., 2022). Although, a recent study reports 3 zinc finger proteins (Bta06175, Bta08766 and Bta11305) that are associated with different pathways and play major roles in *Tomato crinivirus* (ToCV) transmission, to date no study describes the direct interaction of Asia II 7-*BTB*/*POZ* with CLCuMuV-*AV1* and its implication in the virus transmission. Our results demonstrate that CLCuMuV directly targets and differentially regulates the expression of *BTB*/*POZ* gene along with other genes associated with key immunity-related pathways. Insects deploy the innate immune system as the first line of defense to combat invading pathogens which is reflected by the rapid activation of immune responses upon pathogen recognition (Yamamoto-Hino et al., 2015). Implications of the *Jak*/*STAT* and *Toll*-mediated signaling in the insect antiviral immune responses have been highlighted earlier (Dostert et al., 2005; Kingsolver et al., 2013). Interestingly, the findings of a recent study suggest that an RNA genome-containing plant virus (*Rice stripe virus*, RSV) can activate *Toll* signaling pathway in its insect vector *Laodelphax striatellus* (He et al., 2021). While insect’s antiviral immune responses have been well-explored in model insects like mosquitoes and *Drosophila*; they remain unclear in the case of whiteflies. Moreover, understanding of the dynamics of immunity-related signal transduction pathways in response to DNA viruses remains limited. Our findings provide direct evidence that CLCuMuV rapidly activates the antiviral immune responses (*Toll*, *Imd*, *Jnk* and *Jak*/*STAT*) in whiteflies at the early stage of infection. Importantly, the extent of immunity activation remains cryptic species-dependent (**Figure 7**). In future, it would be interesting to explore whether viral AV1 directly interacts with any of these immunity-related factors and hijacks them to facilitate virus accumulation in the insect vector. Given that, begomoviruses are transmitted with variable efficiencies by different whitefly species (Fiallo-Olivé et al., 2020; Wang and Blanc, 2021), and that begomoviruses might selectively interact and regulate host proteins to enhance transmission efficiencies (Wei et al., 2014; Guo et al., 2018; Chi et al., 2021), it would be imperative to investigate whether begomoviruses (or other viruses) recruit *BTB*/*POZ* to successfully overcome/cross various physical barrier/s of the whiteflies.

Apart from the role of *BTB*/*POZ* and antiviral immunity-related genes in viral accumulation, our study also identified additional proteins that putatively interacted with CLCuMuV-AV1. It will be worth researching whether these proteins regulate the differential transmission of the virus by whiteflies. For instance, cytochrome B5 (CsCYTB5) and ribosome-associated proteins of *L. striatellus* have been identified to interact with nucleocapsid protein (pc3) of RSV (Liu et al., 2015). Likewise, a thioredoxin-like whitefly protein has been reported to interact with AV1 of two begomoviruses; *cotton leaf curl Rajasthan virus* (CLCuRaV) and *tomato leaf curl New Delhi virus* (ToLCNDV) (Saurav et al., 2019). However, whether these proteins are directly involved in insect-mediated virus transmission remains a question. Furthermore, eukaryotic translation elongation factors (eEFs) have also been reported to interact with replication-associated proteins p92^pol^ and p33 of a plant-infecting RNA virus (Li et al., 2009). On the other hand, the interaction of eEFs with human DNA viruses has been reported suggesting their vital role in the transcription, replication and DNA synthesis of viral genomes (Yue et al., 2011; Lin et al., 2012). Nevertheless, the putative role of eEFs with regard to whitefly-transmitted, plant DNA viruses completely remains unknown.

In addition to other proteins, our current study has also identified endosymbionts-related protein sequences that interacted with CLCuMuV-AV1. The endosymbiotic bacteria of insects are reportedly known to regulate the host immune responses against pathogenic infections (Eleftherianos et al., 2013). For example, *Wolbachia* is a well-studies endosymbiotic organism that is known to infect over 20% of insect species. Two independent studies have demonstrated that the mutualistic presence of *Wolbachia* in *Drosophila* confers resistance against RNA viruses (Hedges et al., 2008; Teixeira et al., 2008). However, the level of *Wolbachia*-mediated antiviral protection largely depends on the strain selection (Martinez et al., 2014). Furthermore, bacterial symbionts are known to be involved in regulating plant-virus-vector and/or virus-vector interactions. Recently, researchers have demonstrated that a direct interaction between BtR242 (secretory protein of *Rickettsia*) and the coat protein of CLCuMuV facilitates whitefly-borne viral transmission (Lei et al., 2021). These endosymbionts regulate the insect-mediated virus dissemination by affecting the acquisition, stability, release (during viral circulation) and vertical transmission of the virus particles (Wu et al., 2022). Thus, it would be interesting to investigate how these bacterial symbionts govern the variability in virus acquisition and transmission by different biotypes/cryptic species of whiteflies and other insects. Also, exploration and manipulation of these symbionts will help to establish new exciting avenues for the management of plant viral diseases.

Recently, the use of modern, powerful and non-transgenic technologies like clustered regularly interspaced short palindromic repeats (CRISPR)/CRISPR-associated genes (Cas) and RNAi has revolutionized different sectors of plant research (Touzdjian Pinheiro Kohlrausch Távora et al., 2022). For instance, using RNAi to develop transgenic plants to combat geminiviruses and their insect vector have been proposed in the last decade (Kumar and Sarin, 2013). Likewise, researchers have demonstrated that CRISPR/Cas9 confers molecular immunity against multiple geminiviruses (Ali et al., 2016). Several successful cases of using RNAi-mediated targeting of insect genes that transmit plant viruses suggest that RNAi could be a promising technology to control insect pests and especially those that are well-known to facilitate plant virus dissemination (Kanakala and Ghanim, 2016). Thus, in addition to the identification of key/candidate genes that play vital roles in insect-mediated virus transmission, the use of aforementioned technologies to engineer antiviral resistance in crop plants and/or to target host susceptibility genes to prevent insect-borne dissemination of viruses will significantly advance the efforts to attain sustainable management of destructive plant viruses and their vectoring insects.

## Conclusion

**Figure 9:**
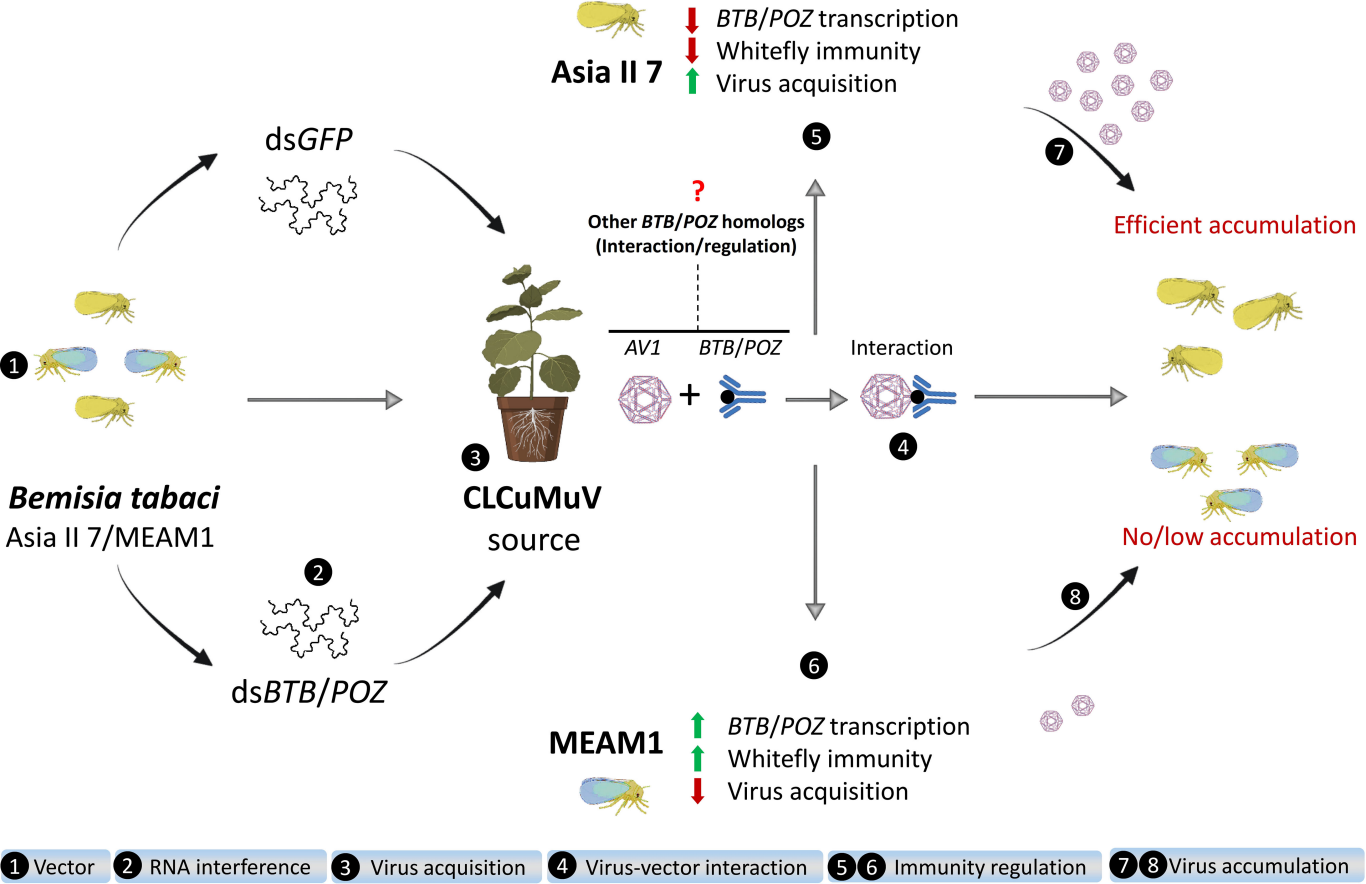
A schematic diagram depicting experimental procedures and the role of CLCuMuV in differential regulation of whitefly antiviral immunity.

Our findings provide compelling evidence that viral determinant *AVI* of a plant begomovirus CLCuMuV targets the innate antiviral immunity of its insect vector *B. tabaci* by directly interacting with BTB/POZ protein and subsequently induces multiple defense-related signaling pathways. Strikingly, this viral targeting and manipulation of the vector immunity take place in a cryptic species-specific manner which consequently regulates differential efficiencies of virus acquisition by Asia II 7 and MEAM1 whiteflies (**Figure 9**). To our knowledge, this is the first study that identifies BTB/POZ as a cellular target of the viral coat protein and highlights its immunity-related role in begomovirus-whitefly interactions. It is worth investigating how these transcriptional repressors regulate virus-insect interactions where the vector carries multiple begomoviruses or viruses from other groups. Additionally, we have identified several candidate proteins of the whitefly vector that putatively interact with viral AV1 factor. Whether these proteins are directly associated with variable virus transmission by different whitefly cryptic species remains a question worth studying in the future. The outcomes of our study will supplement the existing knowledge of virus-vector interactions, the understanding of which is imperative for the timely detection and sustainable management of insect vector-associated viral epidemics.

## Data availability statement

The datasets presented in this study can be found in online repositories. The names of the repository/repositories and accession number(s) can be found in the article/**Supplementary Material**.

## Author contributions

ZH and TF conceived and designed the study. TF, QL, TC, ZL, LY, and GL performed experiments, acquired data and completed formal analysis. TF, YT, and XS prepared the original draft. ZH reviewed and edited the manuscript, supervised and acquired the funding. All authors have read and agreed to the published version of the manuscript.

## Funding

This work was funded by the National Natural Science Foundation of China (31871937, 32001973), Guangdong Basic and Applied Basic Research Foundation (2019A1515012150), the President Foundation of Guangdong Academy of Agricultural Sciences, China (Grant No: BZ202005) and Discipline team building projects of Guangdong Academy of Agricultural Sciences in the 14^th^ Five-Year Period (202105TD).

## Conflict of interest

The authors declare that the research was conducted in the absence of any commercial or financial relationships that could be construed as a potential conflict of interest.

## Publisher’s note

All claims expressed in this article are solely those of the authors and do not necessarily represent those of their affiliated organizations, or those of the publisher, the editors and the reviewers. Any product that may be evaluated in this article, or claim that may be made by its manufacturer, is not guaranteed or endorsed by the publisher.
