# Supplementary material for: Cotton leaf curl Multan virus differentially regulates innate antiviral immunity of whitefly (Bemisia tabaci) vector to promote cryptic species-dependent virus acquisition
Source: Front Plant Sci. 2022 Nov 14;13:1040547. doi: 10.3389/fpls.2022.1040547 (PMC9702342; doi:10.3389/fpls.2022.1040547)
Supplement: Supplementary file 1 [file DataSheet_1.docx]

## Supplementary Figures

**
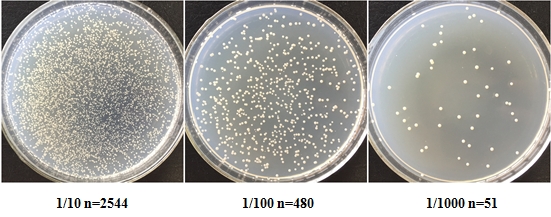
**

**Supplementary Figure S1.** Transformation efficiency Asia II 7 cDNA library screened against CLCuMuV-AV1.

**
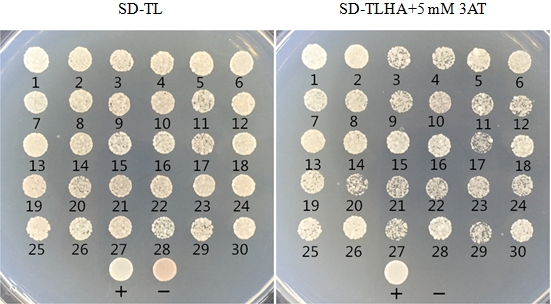
**

**Supplementary Figure S2.** Detection of ade2 and his3 reporter genes activated by initially positive clones.

**
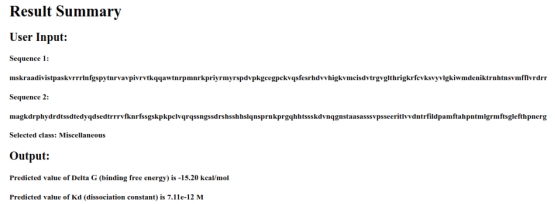
**

**Supplementary Figure S3.** Prediction of protein-protein interaction between CLCuMuV-AV1 (sequence 1) and BTB/POZ (sequence 2) based on the Gibbs free energy ΔΔG score.

**Supplementary Table S1:** Details of primers used in this study

| Primer | Sequence (5'-3') | Product size (bp) | Purpose | Reference |
| --- | --- | --- | --- | --- |
| mtCOI-C1- J- 2195 | TTGATTTTTTGGTCATCCAGAAGT | 880 | biotype verification | [1] |
| mtCOI-TL2N- 3014 | TCCAATGCACTAATCTGCCATATTA |  |  |  |
| CLCuMuV-CL-F | CAGGAAGCAGGAAAATACGAGA | 831 | CLCuMuV detection | [2] |
| CLCuMuV-CL-R | TGGCAGTCCAACACAAAATACG |  |  |  |
| CLCuMuB-beta-F | AAGTCGAATGGAACGTGAATGT | 837 | CLCuMuV detection | [2] |
| CLCuMuB-beta-R | GGAGACCAAAAGAGGAGAGAGA |  |  |  |
| Y2Hb-AV1-F | AAGGCCATTACGGCCATGTCGAAGCGAGCTGCAGA | 768 | yeast two-hybrid | present study |
| Y2Hb-AV1-R | CCGGCCGAGGCGGCCCCATTCGTTACAGAGTCATAAA |  |  |  |
| Y2Hp-BTB-F | AAGGCCATTACGGCCATGGCTGGAAAAGACAGACC | 1248 | yeast two-hybrid | present study |
| Y2Hp-BTB-R | CCGGCCGAGGCGGCCAGGATCTTCATGAGCCGC |  |  |  |
| BFCBTB-F | CGGACTAGTATGGCTGGAAAAGACAGACC | 1248 | BiFC | present study |
| BFCBTB-R | ACGCGTCGACAGGATCTTCATGAGCCGC |  |  |  |
| BFCCP-F | CGGACTAGTATGTCGAAGCGAGCTGCAGA | 771 | BiFC | present study |
| BFCCP-R | ACGCGTCGACATTCGTTACAGAGTCATA |  |  |  |
| T7BTBRi-F | GGATCCTAATACGACTCACTATAGGATGGCTGGAAAAGACAGACC | 405 | RNAi | present study |
| T7BTBRi-R | GGATCCTAATACGACTCACTATAGGGGTGAACATTCTGCCCAACA |  |  |  |
| T7GFPRi-F | TAATACGACTCACTATAGGGCAGTGGAGAGGGTGAA | 310 | RNAi | present study |
| T7GFPRi-R | TAATACGACTCACTATAGGGTTGACGAGGGTGTCTC |  |  |  |
| qpBtActin-F | TCTTCCAGCCATCCTTCTTG | 120 | qPCR | present study |
| qpBtActin-R | CGGTGATTTCCTTCTGCATT |  |  |  |
| qpAV1-F | GGCTTTGGTCAAGAAGTTTGTC | 120 | qPCR | present study |
| qpAV1-R | GCGTGGGTACAAGCCATATAA |  |  |  |
| qpBTB-F | GGACTCGAATTCACACATCCTAA | 120 | qPCR | present study |
| qpBTB-R | GGGTGGACAACGGATGATTT |  |  |  |
| qpBta11797-F | CTGTGGTTTGTTACGGAGGA | 120 | qPCR | present study |
| qpBta11797-R | GCCACTTTCCAACTGAGGTA |  |  |  |
| qpBta12916-F | GCTCAAGCTGTCGTCGAATA | 120 | qPCR | present study |
| qpBta12916-R | AACACATCAGGGTGCAGTC |  |  |  |
| qpBta14468-F | ACAGTACTGAGTTAGGGCTTTC | 120 | qPCR | present study |
| qpBta14468-R | CGGAGGGAATATCAACCACTATC |  |  |  |
| qpBta12796-F | ACAGAGCGTACATTGACAAGAG | 120 | qPCR | present study |
| qpBta12796-R | CTGCCAGGAGTCACGTAAAC |  |  |  |
| qpBta12398-F | TTGGAAACAAAGGGCATTTCAG | 120 | qPCR | present study |
| qpBta12398-R | CGAACGGTTCCACCATGAATA |  |  |  |
| qpBta08531-F | CGGAAAGTCCTCCGAAAGTAAG | 120 | qPCR | present study |
| qpBta08531-R | CAGCTGGATTGTTAGCGTCA |  |  |  |
| qpBta12850-F | CCGGCACCTATTTGGTCTT | 120 | qPCR | present study |
| qpBta12850-R | CTGGGCACCTTGAACCTAAT |  |  |  |
| qpBta03387-F | AACACTACATGCTGATCTGTCC | 120 | qPCR | present study |
| qpBta03387-R | CCACTTAGCTGTTGGTCTCTTT |  |  |  |

**Supplementary Table S2:** Combinations of plasmids used for Y2H screening experiments

| **Reaction** | **AD plasmid** | **BD plasmid** | **Selective media** | **Purpose** |
| --- | --- | --- | --- | --- |
| **1** | pNubG-Fe65 | pTSU2-APP | SD-TL、SD-TLH、SD-TLHA | Positive control |
| **2** | pPR3N | pTSU2-APP | SD-TL、SD-TLH、SD-TLHA | Negative control |
| **3** | pPR3N | pBT3STE-AV1 | SD-TL、SD-TLH、SD-TLHA | Self-activation test |
| **4** | pPR3N | pBT3SUC-AV1 | SD-TL、SD-TLH、SD-TLHA | Self-activation test |
| **5** | pOST1-NubI | pBT3STE-AV1 | SD-TL、SD-TLH、SD-TLHA | Functional test |
| **6** | pOST1-NubI | pBT3SUC-AV1 | SD-TL、SD-TLH、SD-TLHA | Functional test |
| **7** | ———— | pBT3STE-AV1 | SD-L | Bait preservation |
| **8** | ———— | pBT3SUC-AV1 | SD-L | Bait preservation |

**Supplementary Table S3:** BTB/POZ domain-containing proteins from different insect species used in the phylogenetic analysis

| No. | GenBank accession | Description | Scientific name | Identity (%) | Protein size (aa) |
| --- | --- | --- | --- | --- | --- |
|  | XP_018901332.1 | PREDICTED: BTB/POZ domain-containing protein 10 isoform X2 | *Bemisia tabaci* | 100 | 415 |
|  | XP_018901329.1 | PREDICTED: BTB/POZ domain-containing protein 10 isoform X1 | *Bemisia tabaci* | 96.51 | 430 |
|  | XP_021936321.1 | BTB/POZ domain-containing protein 10 isoform X3 | *Zootermopsis nevadensis* | 77.51 | 430 |
|  | XP_021936320.1 | BTB/POZ domain-containing protein 10 isoform X2 | *Zootermopsis nevadensis* | 77.51 | 436 |
|  | XP_021936319.1 | BTB/POZ domain-containing protein 10 isoform X1 | *Zootermopsis nevadensis* | 77.51 | 477 |
|  | RZF48082.1 | hypothetical protein LSTR_LSTR002148 | *Laodelphax striatellus* | 78.88 | 487 |
|  | XP_014244086.1 | BTB/POZ domain-containing protein 10 | *Cimex lectularius* | 76.59 | 404 |
|  | XP_024219824.1 | BTB/POZ domain-containing protein 10 | *Halyomorpha halys* | 75.87 | 401 |
|  | XP_018569823.1 | BTB/POZ domain-containing protein 10 isoform X1 | *Anoplophora glabripennis* | 76.21 | 455 |
|  | GFG28770.1 | hypothetical protein Cfor_03009 | *Coptotermes formosanus* | 75.29 | 446 |
|  | XP_022919872.1 | BTB/POZ domain-containing protein 10-like isoform X1 | *Onthophagus taurus* | 74.4 | 452 |
|  | XP_023704255.1 | BTB/POZ domain-containing protein 10 isoform X4 | *Cryptotermes secundus* | 78.93 | 436 |
|  | XP_023704254.2 | BTB/POZ domain-containing protein KCTD20 isoform X1 | *Cryptotermes secundus* | 78.93 | 481 |
|  | XP_006559059.1 | uncharacterized protein LOC409323 | *Apis mellifera* | 71.5 | 1442 |
|  | XP_016657952.1 | BTB/POZ domain-containing protein 10 isoform X1 | *Acyrthosiphon pisum* | 71.84 | 448 |
|  | XP_001951382.1 | BTB/POZ domain-containing protein 10 isoform X2 | *Acyrthosiphon pisum* | 71.84 | 444 |
|  | XP_031772411.1 | protein nervous wreck | *Apis florea* | 71.5 | 1443 |
|  | XP_029054034.1 | LOW QUALITY PROTEIN: protein nervous wreck-like | *Osmia bicornis bicornis* | 71.26 | 1511 |
|  | XP_026808407.1 | BTB/POZ domain-containing protein 10 | *Rhopalosiphum maidis* | 71.78 | 443 |
|  | XP_034190892.1 | LOW QUALITY PROTEIN: protein nervous wreck-like | *Osmia lignaria* | 71.26 | 1517 |
|  | VEN55356.1 | unnamed protein product | *Callosobruchus maculatus* | 76.36 | 453 |
|  | XP_017794172.1 | PREDICTED: LOW QUALITY PROTEIN: uncharacterized protein LOC108575790 | *Habropoda laboriosa* | 71.26 | 1437 |
|  | KAF5286728.1 | hypothetical protein FQR65_LT12461 | *Abscondita terminalis* | 73.65 | 478 |
|  | XP_018056251.1 | PREDICTED: uncharacterized protein LOC108692489 | *Atta colombica* | 74.87 | 1352 |
|  | XP_018366748.1 | PREDICTED: uncharacterized protein LOC108763530 | *Trachymyrmex cornetzi* | 74.43 | 1400 |
|  | XP_033303947.1 | LOW QUALITY PROTEIN: protein nervous wreck-like | *Bombus bifarius* | 70.71 | 1445 |
|  | XP_033183202.1 | LOW QUALITY PROTEIN: protein nervous wreck-like | *Bombus vancouverensis nearcticus* | 70.71 | 1445 |
|  | XP_016911115.1 | BTB/POZ domain-containing protein KCTD20 isoform X1 | *Apis cerana* | 71.43 | 523 |
|  | XP_022183575.1 | BTB/POZ domain-containing protein 10 | *Myzus persicae* | 72.57 | 443 |
|  | KAG5328714.1 | BTBDA protein | *Acromyrmex charruanus* | 73.57 | 1419 |
|  | XP_018355590.1 | PREDICTED: uncharacterized protein LOC108756342 | *Trachymyrmex septentrionalis* | 74.43 | 1399 |
|  | KAF0770767.1 | BTB/POZ domain-containing protein 10 | *Aphis craccivora* | 70.17 | 453 |
|  | XP_026274530.1 | BTB/POZ domain-containing protein KCTD20-like | *Frankliniella occidentalis* | 76.53 | 546 |
|  | KAE9539428.1 | hypothetical protein AGLY_004680 | *Aphis glycines* | 71.05 | 437 |
|  | KOX78425.1 | BTB/POZ domain-containing protein 10 | *Melipona quadrifasciata* | 71.26 | 478 |
|  | KYQ47590.1 | BTB/POZ domain-containing protein 10 | *Trachymyrmex zeteki* | 74.42 | 483 |
|  | KYN44081.1 | BTB/POZ domain-containing protein 10 | *Trachymyrmex septentrionalis* | 74.43 | 442 |
|  | KYN17192.1 | BTB/POZ domain-containing protein 10 | *Trachymyrmex cornetzi* | 74.43 | 442 |
|  | XP_028141132.1 | BTB/POZ domain-containing protein 10 isoform X1 | *Diabrotica virgifera virgifera* | 72.49 | 449 |
|  | XP_027837542.1 | BTB/POZ domain-containing protein 10 | *Aphis gossypii* | 70.8 | 437 |
|  | XP_025194895.1 | BTB/POZ domain-containing protein 10 | *Melanaphis sacchari* | 71.82 | 443 |
|  | KOC61530.1 | BTB/POZ domain-containing protein 10 | *Habropoda laboriosa* | 71.19 | 478 |
|  | XP_018403716.1 | PREDICTED: uncharacterized protein LOC108780484 | *Cyphomyrmex costatus* | 73.55 | 1401 |
|  | XP_011688351.1 | PREDICTED: BTB/POZ domain-containing protein 10 isoform X3 | *Wasmannia auropunctata* | 75.06 | 449 |
|  | XP_019878498.1 | PREDICTED: BTB/POZ domain-containing protein 10-like isoform X1 | *Aethina tumida* | 75.71 | 464 |
|  | XP_012152987.1 | PREDICTED: BTB/POZ domain-containing protein 10 isoform X2 | *Megachile rotundata* | 70.09 | 507 |
|  | XP_012152989.1 | PREDICTED: BTB/POZ domain-containing protein KCTD20 isoform X3 | *Megachile rotundata* | 70.78 | 500 |
|  | XP_011056181.1 | PREDICTED: BTB/POZ domain-containing protein 10 isoform X2 | *Acromyrmex echinatior* | 74.55 | 438 |
|  | XP_015429856.1 | PREDICTED: BTB/POZ domain-containing protein 10 isoform X3 | *Dufourea novaeangliae* | 71.15 | 453 |
|  | XP_015429853.1 | PREDICTED: BTB/POZ domain-containing protein KCTD20 isoform X1 | *Dufourea novaeangliae* | 71.15 | 494 |
|  | XP_011056180.1 | PREDICTED: BTB/POZ domain-containing protein 10 isoform X1 | *Acromyrmex echinatior* | 74.55 | 446 |
|  | XP_012165141.1 | BTB/POZ domain-containing protein 10 isoform X2 | *Bombus terrestris* | 70.64 | 470 |
|  | XP_019872106.1 | PREDICTED: BTB/POZ domain-containing protein 10-like | *Aethina tumida* | 75.71 | 441 |
|  | XP_012237914.1 | BTB/POZ domain-containing protein KCTD20 isoform X1 | *Bombus impatiens* | 70.64 | 477 |
|  | XP_018327198.1 | BTB/POZ domain-containing protein 10 isoform X2 | *Agrilus planipennis* | 76.67 | 465 |
|  | XP_024221908.1 | BTB/POZ domain-containing protein 10 isoform X3 | *Bombus impatiens* | 70.64 | 434 |
|  | XP_017778022.1 | PREDICTED: BTB/POZ domain-containing protein 10 isoform X1 | *Nicrophorus vespilloides* | 73.45 | 442 |
|  | XP_034255850.1 | BTB/POZ domain-containing protein 10-like | *Thrips palmi* | 73.87 | 594 |
|  | XP_011688350.1 | PREDICTED: BTB/POZ domain-containing protein 10 isoform X2 | *Wasmannia auropunctata* | 73.18 | 452 |
|  | XP_018327197.1 | BTB/POZ domain-containing protein 10 isoform X1 | *Agrilus planipennis* | 76.28 | 467 |
|  | CAD7571615.1 | unnamed protein product | *Timema californicum* | 77.95 | 453 |
|  | XP_012542451.1 | BTB/POZ domain-containing protein 10 isoform X1 | *Monomorium pharaonis* | 73.42 | 442 |
|  | KZC08283.1 | BTB/POZ domain-containing protein 10 | *Dufourea novaeangliae* | 69.72 | 504 |
|  | XP_011251338.2 | BTB/POZ domain-containing protein 10 isoform X1 | *Camponotus floridanus* | 70.57 | 535 |
|  | XP_015124869.1 | BTB/POZ domain-containing protein KCTD20 isoform X1 | *Diachasma alloeum* | 71.66 | 476 |
|  | XP_011874501.1 | PREDICTED: BTB/POZ domain-containing protein 10 isoform X1 | *Vollenhovia emeryi* | 73.42 | 442 |
|  | XP_011343954.1 | BTB/POZ domain-containing protein 10 | *Ooceraea biroi* | 69.91 | 449 |
|  | CAD7393447.1 | unnamed protein product | *Timema cristinae* | 77.69 | 444 |
|  | XP_011644138.1 | BTB/POZ domain-containing protein 10 isoform X1 | *Pogonomyrmex barbatus* | 73.67 | 442 |
|  | CAD7453196.1 | unnamed protein product | *Timema tahoe* | 77.69 | 454 |
|  | EFN71747.1 | BTB/POZ domain-containing protein 10 | *Camponotus floridanus* | 70.57 | 499 |
|  | VVC40483.1 | BTB/POZ domain,SKP1/BTB/POZ domain | *Cinara cedri* | 72.08 | 447 |
|  | CAD7445302.1 | unnamed protein product | *Timema bartmani* | 77.69 | 477 |
|  | XP_031346632.1 | BTB/POZ domain-containing protein KCTD20 isoform X1 | *Photinus pyralis* | 70.5 | 465 |
|  | CAD7593558.1 | unnamed protein product | *Timema genevievae* | 77.69 | 464 |
|  | XP_019753821.1 | PREDICTED: BTB/POZ domain-containing protein 10 isoform X2 | *Dendroctonus ponderosae* | 68.82 | 426 |
|  | XP_043286760.1 | BTB/POZ domain-containing protein 10 isoform X2 | *Venturia canescens* | 73.4 | 448 |
|  | XP_035723326.1 | BTB/POZ domain-containing protein 10-like isoform X3 | *Vespa mandarinia* | 70.41 | 435 |
|  | XP_043286759.1 | BTB/POZ domain-containing protein KCTD20 isoform X1 | *Venturia canescens* | 69.23 | 479 |
|  | XP_035723318.1 | BTB/POZ domain-containing protein KCTD20-like isoform X1 | *Vespa mandarinia* | 70.41 | 476 |

**Supplementary Table S4:** Attributes of AV1-interacting whitefly proteins identified by Y2H screening

| No. | Y2H identified (NCBI Accession) | Protein Accession | GO Term | Category* | Function | Type | Interpro ID |
| --- | --- | --- | --- | --- | --- | --- | --- |
| 1 | XM_025948751.1 | Bta03911 | GO:0005515  GO:0043167 | MF | Protein binding  Ion binding | Chaperone protein dnaj | IPR001305 |
| 2 | XM_015784844.2 | Bta13632 | GO:0002168  GO:0005739  GO:0005811  GO:0006118  GO:0009055  GO:0012505  GO:0016020  GO:0035206  GO:0045610  GO:0046872  GO:0051726 | MF BF  CC | instar larval development(biological_process)  mitochondrion(cellular_component)  lipid particle(cellular_component)  obsolete electron transport(biological_process)  electron carrier activity(molecular_function)  endomembrane system(cellular_component)  membrane(cellular_component)  regulation of hemocyte proliferation(biological_process)  regulation of hemocyte differentiation(biological_process)  metal ion binding(molecular_function)  regulation of cell cycle(biological_process) | Cytochrome B5 | IPR001199 |
| 3 | KM821541.1 | Bta01987 | GO:0004129  GO:0005506  GO:0005743  GO:0006123  GO:0009060  GO:0016021  GO:0020037  GO:0045277  GO:1902600 | MF BF  CC | cytochrome-c oxidase activity(molecular_function)  iron ion binding(molecular_function)  mitochondrial inner membrane(cellular_component)  mitochondrial electron transport, cytochrome c to oxygen(biological_process)  aerobic respiration(biological_process)  integral component of membrane(cellular_component)  heme binding(molecular_function)  respiratory chain complex IV(cellular_component)  hydrogen ion transmembrane transport(biological_process) | Cytochrome c oxidase subunit 1 | IPR000883 |
| 4 | FP100145.1 | Bta15795 | GO:0003676  GO:0004386  GO:0005524  GO:0008152 | BF  MF | nucleic acid binding(molecular_function)  helicase activity(molecular_function)  ATP binding(molecular_function)  metabolic process(biological_process) | ATP-dependent RNA helicase | IPR000629 |
| 5 | XM_019048796.1 | Bta00134 | GO:0003746  GO:0003924  GO:0005525  GO:0005840  GO:0006448 | MF BF  CC | translation elongation factor activity(molecular_function)  GTPase activity(molecular_function)  GTP binding(molecular_function)  ribosome(cellular_component)  regulation of translational elongation(biological_process) | Elongation factor 2 | IPR000640 |
| 6 | XM_003557410.4 | Bta12552 | GO:0005634  GO:0006118  GO:0006662  GO:0006974  GO:0006979  GO:0008340  GO:0015035  GO:0045454  GO:0050832  GO:0055114 | MF BF  CC | nucleus(cellular_component)  obsolete electron transport(biological_process)  glycerol ether metabolic process(biological_process)  cellular response to DNA damage stimulus(biological_process)  response to oxidative stress(biological_process)  determination of adult lifespan(biological_process)  protein disulfide oxidoreductase activity(molecular_function)  cell redox homeostasis(biological_process)  defense response to fungus(biological_process)  oxidation-reduction process(biological_process) | Thioredoxin | IPR005746 |
| 7 | FP099701.1 | Bta10027 | - | - | - | Unknown protein | - |
| 8 | XM_015785310.2 | Bta03336 | GO:0006118  GO:0006662  GO:0015035  GO:0045454  GO:0055114 | MF  BF | obsolete electron transport(biological_process)  glycerol ether metabolic process(biological_process)  protein disulfide oxidoreductase activity(molecular_function)  cell redox homeostasis(biological_process)  oxidation-reduction process(biological_process) | Thioredoxin-like protein 1 | IPR005746 |
| 9 | FP094029.1 | Bta13336 | GO:0000786  GO:0003677  GO:0005634  GO:0046982 | MF CC | nucleosome(cellular_component)  DNA binding(molecular_function)  nucleus(cellular_component)  protein heterodimerization activity(molecular_function) | Histone H2A | IPR002119 |
| 10 | CP016327.1 | - |  |  |  | Candidatus Portiera aleyrodidarum strain BT-Z1 | - |
| 11 | XM_020323159.1 | Bta13422 | GO:0003924  GO:0005200  GO:0005525  GO:0005737  GO:0005874  GO:0006184  GO:0016203  GO:0051258  GO:0051298 | MF BF  CC | GTPase activity(molecular_function)  structural constituent of cytoskeleton(molecular_function)  GTP binding(molecular_function)  cytoplasm(cellular_component)  microtubule(cellular_component)  obsolete GTP catabolic process(biological_process)  muscle attachment(biological_process)  protein polymerization(biological_process)  centrosome duplication(biological_process) | Tubulin beta-1 chain | IPR000217 |
| 12 | KX714967.1 | - | - | - | - | Bemisia emiliae mitochondrion, partial genome | - |
| 13 | FP100486.1 | Bta10819 | - | - | - | Unknown protein | - |
| 14 | XM_003571129.4 | Bta07620 | GO:0003677  GO:0004674  GO:0005524  GO:0005634  GO:0006468  GO:0009069 | MF BF  CC | DNA binding(molecular_function)  protein serine/threonine kinase activity(molecular_function)  ATP binding(molecular_function)  nucleus(cellular_component)  protein phosphorylation(biological_process)  serine family amino acid metabolic process(biological_process) | High mobility group protein B2, putative | IPR009071 |
| 15 | CP016343.1 | - |  |  |  | Candidatus Portiera aleyrodidarum strain China 1 | - |
| 16 | CP007563.1 | - |  |  |  | Candidatus Portiera aleyrodidarum MED (*Bemisia tabaci*) strain BT-Q | - |
| 17 | XM_020325952.1 | Bta12951 | - | - | - | Unknown protein | - |
| 18 | FP100540.1 | Bta13313 | GO:0000022  GO:0003743  GO:0005829  GO:0005840  GO:0006446  GO:0006909  GO:0007224  GO:0016282 | MF BF  CC | mitotic spindle elongation(biological_process)  translation initiation factor activity(molecular_function)  cytosol(cellular_component)  ribosome(cellular_component)  regulation of translational initiation(biological_process)  phagocytosis(biological_process)  smoothened signaling pathway(biological_process)  eukaryotic 43S preinitiation complex(cellular_component) | Eukaryotic translation initiation factor 1A | IPR001253 |
| 19 | XM_019045788.1 | Bta12389 | GO:0005515 | MF | Protein binding | BTB/POZ domain-containing protein 10 | IPR000210 |
| 20 | XM_015791561.2 | Bta08869 | GO:0005789  GO:0008152  GO:0016021  GO:0016740 | MF  BF  CC | endoplasmic reticulum membrane(cellular_component)  metabolic process(biological_process)  integral component of membrane(cellular_component)  transferase activity(molecular_function) | Oligosaccaryltransferase | IPR018943 |
| 21 | FP096515.1 | Ssa13476 | - | - | - | CG13675, isoform D | - |

*MF=molecular function, BF= biological function, CC= cellular component

**Supplementary Table S5:** *Bemisia tabaci* genes associated with different antiviral immunity-related pathways

| **Gene ID** | **Gene name** | **Pathway** | **Size (bp)** |
| --- | --- | --- | --- |
| Bta11797 | Toll-like receptor | TOLL | 3153 |
| Bta12916 | Toll receptor 3 | TOLL | 3831 |
| Bta14468 | FAS-associated factor 1 | IMD | 1968 |
| Bta12796 | Serine/threonine-protein kinase TBK1 | IMD | 738 |
| Bta12398 | JNK1/MAPK8-associated membrane protein | JNK/MAPK | 900 |
| Bta08531 | C-jun-amino-terminal kinase-interacting protein | JNK/RAB | 3198 |
| Bta12850 | Tyrosine-protein kinase | JAK/STAT | 3417 |
| Bta03387 | Signal transducer and activator of transcription | JAK/STAT | 2394 |

**Supplementary Table S6:** Comparative analysis of BTB/POZ domain-containing genes among CLCuMuV-infected MEAM1 and Asia II 7 whiteflies (*Bemisia tabaci*)

| **No.** | **Gene ID** | **Mean FPKM values after CLCuMuV infection** | | **Difference** | **Significant? (yes/no)** | ***P*-value** |
| --- | --- | --- | --- | --- | --- | --- |
|  |  | **MEAM1** | **Asia II 7** |  |  |  |
|  | Bta03927 | 1.227 | 0.6633 | 0.5633 | Yes | 0.005054 |
|  | Bta03430 | 1.073 | 2.003 | -0.9300 | Yes | 0.003290 |
|  | Bta12389 | 31.03 | 35.16 | -4.137 | No | 0.143640 |
|  | Bta05621 | 5.663 | 6.363 | -0.7000 | No | 0.032556 |
|  | Bta10433 | 4.277 | 4.203 | 0.07333 | No | 0.879875 |
|  | Bta11335 | 60.14 | 11.53 | 48.61 | Yes | 0.000342 |
|  | Bta11527 | 7.047 | 6.183 | 0.8633 | No | 0.356501 |
|  | Bta05204 | 10.07 | 3.347 | 6.720 | Yes | 0.011922 |
|  | Bta11528 | 10.52 | 4.787 | 5.730 | Yes | 0.000757 |
|  | Bta05682 | 1.330 | 7.623 | -6.293 | Yes | 0.000193 |
|  | Bta07533 | 18.88 | 1.910 | 16.97 | Yes | 0.000010 |
|  | Bta00558 | 0.6833 | 0.3600 | 0.3233 | No | 0.027124 |
|  | Bta11936 | 11.05 | 14.07 | -3.027 | No | 0.022386 |
|  | Bta13385 | 0.8967 | 2.703 | -1.807 | Yes | 0.000447 |
|  | Bta00956 | 4.063 | 7.090 | -3.027 | Yes | 0.005337 |
|  | Bta07534 | 0.3467 | 0.1200 | 0.2267 | No | 0.426517 |
|  | Bta07575 | 2.430 | 3.377 | -0.9467 | Yes | 0.002388 |
|  | Bta11336 | 4.443 | 1.163 | 3.280 | Yes | 0.011313 |
|  | Bta11337 | 0.09333 | 0.9333 | -0.8400 | Yes | 0.000497 |
|  | Bta11141 | 1.330 | 2.083 | -0.7533 | Yes | 0.002507 |
|  | Bta14502 | 5.657 | 2.030 | 3.627 | Yes | 0.000293 |
|  | Bta03712 | 5.040 | 3.613 | 1.427 | No | 0.059481 |
|  | Bta14779 | 14.60 | 10.71 | 3.890 | Yes | 0.000691 |

* The datasets associated with supplementary table S6 can be found in online repositories. The names of the repository/repositories and accession number(s) is:
NCBI, PRJNA865034.

**References:**

[1] Frohlich DR, Torres-Jerez II, Bedford ID, Markham PG, Brown JK. A phylogeographical analysis of the *Bemisia tabaci* species complex based on mitochondrial DNA markers. Molecular ecology. 1999;8(10):1683-91.

[2] Tang YF, He Z, Du ZG, She XM, Lan GB. The Complex of Cotton leaf curl Multan virus and Its Associated Betasatellite Molecule Causing Cotton Leaf Curl Disease in Guangdong Province. Scientia Agricultura Sinica 2015;48(16):3166-75.
